# Tumor microbiome: roles in tumor initiation, progression, and therapy

**DOI:** 10.1186/s43556-025-00248-9

**Published:** 2025-02-08

**Authors:** Shengxin Zhang, Jing Huang, Zedong Jiang, Huan Tong, Xuelei Ma, Yang Liu

**Affiliations:** 1 Department of Biotherapy, Cancer Center and State Key Laboratory of Biotherapy, West China Hospital, Sichuan University, Chengdu, 610041 Sichuan Province China; 2 Department of Medical Ultrasound, West China Hospital of Sichuan University, 37 Guoxue Lane, Wuhou District, Chengdu, 610041 Sichuan Province China; 3 Day Surgery Center, General Practice Medical Center, West China Hospital, Sichuan University, Chengdu, Sichuan 610041 P. R. China

**Keywords:** Tumor microbiome, Tumor microenvironment, Tumor initiation and progression, Immune modulation, Microbe-based therapeutic strategies

## Abstract

Over the past few years, the tumor microbiome is increasingly recognized for its multifaceted involvement in cancer initiation, progression, and metastasis. With the application of 16S ribosomal ribonucleic acid (16S rRNA) sequencing, the intratumoral microbiome, also referred to as tumor-intrinsic or tumor-resident microbiome, has also been found to play a significant role in the tumor microenvironment (TME). Understanding their complex functions is critical for identifying new therapeutic avenues and improving treatment outcomes. This review first summarizes the origins and composition of these microbial communities, emphasizing their adapted diversity across a diverse range of tumor types and stages. Moreover, we outline the general mechanisms by which specific microbes induce tumor initiation, including the activation of carcinogenic pathways, deoxyribonucleic acid (DNA) damage, epigenetic modifications, and chronic inflammation. We further propose the tumor microbiome may evade immunity and promote angiogenesis to support tumor progression, while uncovering specific microbial influences on each step of the metastatic cascade, such as invasion, circulation, and seeding in secondary sites. Additionally, tumor microbiome is closely associated with drug resistance and influences therapeutic efficacy by modulating immune responses, drug metabolism, and apoptotic pathways. Furthermore, we explore innovative microbe-based therapeutic strategies, such as engineered bacteria, oncolytic virotherapy, and other modalities aimed at enhancing immunotherapeutic efficacy, paving the way for microbiome-centered cancer treatment frameworks.

## Introduction

Human microorganisms include bacteria, fungi, viruses, and archaea, they inhabit a diverse range of sites in the human body, particularly the alimentary system, which harbors trillions of microbial communities [1]. The past decades have uncoverred the secret that gut microbiomes can influence immune function and metabolic pathways through specific metabolites and receptors in a diverse range of diseases, especially malignancies [2, 3]. The currently recognized carcinogenic microbiota involve bacteria, fungi, and viruses, among which they increase the susceptibility to tumor through a series of complicated mechanisms, such as chronic inflammation, activation of oncogenic pathways, and genome profile modifications [4, 5]. These tumor microbiomes have been explored to correlate to tumor incidence, progression, and metastasis from multiple steps [6]. Additionally, the specific microflora have been proven with the capability of influencing the efficacy of immunotherapy concurrently, providing the potential of combining standardized tumor treatments with microbiome-based adjuvant therapies [7–10].

TME, once thought to be sterile, has been discovered to harbor specific microorganisms with relatively lower biomass compared with gut microbiome, thanks to advancements in techniques such as 16S rRNA sequencing, metagenomic sequencing, and fluorescence in situ hybridization (FISH)) [11]. These microbial communities that reside in the TME as an intrinsic part are referred to as intratumoral microbiota, also known as the tumor-intrinsic microbiome. The role of intratumoral microbiota has been increasingly recognized, particularly in relation to malignancies [12]. Although emerging studies have begun to explore the crucial function of tumor microbiomes in different malignancies, many issues still remain, such as how they contribute to tumor progression, vary across tumor stages, and affect immunotherapeutic effectiveness. Most importantly, due to the low biomass and the complexity of the extraction process of intratumoral microbiota, distinguishing between the tumor microbiome and the intratumoral microbiome is often challenging. In fact, sometimes the definitions of these two concepts may overlap, as both refer to microbial communities associated with tumors. The former comprise all of the diversified microorganisms around or inside the tumor tissues, with its scope being broader and often including the sources of the latter. The latter, on the other hand, specifically refers to microorganisms only present within the tumor tissues. Therefore, addressing these uncertainties will be essential for fully harnessing the potential of microbiome-targeted strategies in tumor treatment.

This review aims to focus on the intricate relationship between microbiome and tumors. Firstly, we explore the diversity and origins of tumor microbiota across different tumor types. Then we explore how these microorganisms facilitate tumor development and metastasis, including their roles in oncogenic transformation, immune evasion, and TME modification. Moreover, we evaluate how certain microbiomes can mediate chemotherapeutic resistance, while others enhance the efficacy of chemotherapy, radiotherapy, and immunotherapy. Finally, we summarize current challenges and propose future prospects of microbiome-based novel tumor therapies.

## The origin and composition of intratumoral microbiota

### Microbiota entry and establishment within TME

Intratumoral microbiota likely possess various sources, given the high specificity of each tumor-intrinsic bacterial subpopulation [13]. One acknowledged route for microbiota entry is invasion of mucosal barriers. For example, tumors originating from mucosal surfaces, such as colorectal, pancreatic, esophageal, cervical, and lung cancers, often harbor microorganisms [14]. When these mucosal barriers are compromised, they create a pathway for external bacteria to invade tumor tissues [15]. In pancreatic cancer, for instance, the microbiome is primarily thought to come from the gut and oral cavity, of which key subpopulations include *Enterococcus faecalis (E. faecalis)*, *Escherichia coli (E. coli)*, and *Proteobacteria*, likely migrating through the pancreatic duct [16]. Additionally, direct invasion from adjacent tissues is another route, as studies have found a striking similarity between the microbiota in tumors and that in adjacent tissues, suggesting that intratumoral microbiota may arise from the local microbial populations [11]. Evidence includes studies comparing the microbial communities in tumors with those in nearby healthy tissues. Riquelme et al. highlighted significant similarities between intratumoral microbiota and adjacent tissues, and Nejman et al. demonstrated it through 16S rRNA sequencing of various tumor types [11, 17]. Moreover, researchers have increasingly suggested that the circulatory system is also an indispensable path for microorganisms to spread accordingly, by which microorganisms from sites such as the oral cavity, gut, and other locations are transported to tumors and establish themselves within the TME [18]. Pushalkar et al. demonstrated that gut microbiota can translocate via the bloodstream to pancreatic tumors, influencing tumorigenesis by modulating both innate and adaptive immune responses [19]. Besides, Choi Y et al. proposed that immune checkpoint inhibitors (ICIs) administration could induce the microbial transferring by means of secondary lymphoid organs and subcutaneous melanoma tumors, with activating dendritic cells (DCs) activities and enhancing anti-tumor T-cell responses. This finding suggested the possibility of microorganisms originating from the lymphatic system [20]. Also, Bullman S et al. indicated the stability of microbial diversity in both primary and metastatic lesions of colorectal cancer, suggesting the microbial indeed spread through the vascular system [21]. Overall, these findings support the view that tumor-intrinsic microbiota should be regarded as an integral part of the TME, independent of external infections [22].

### Diversity of microbial species across tumor types

**Fig. 1:**
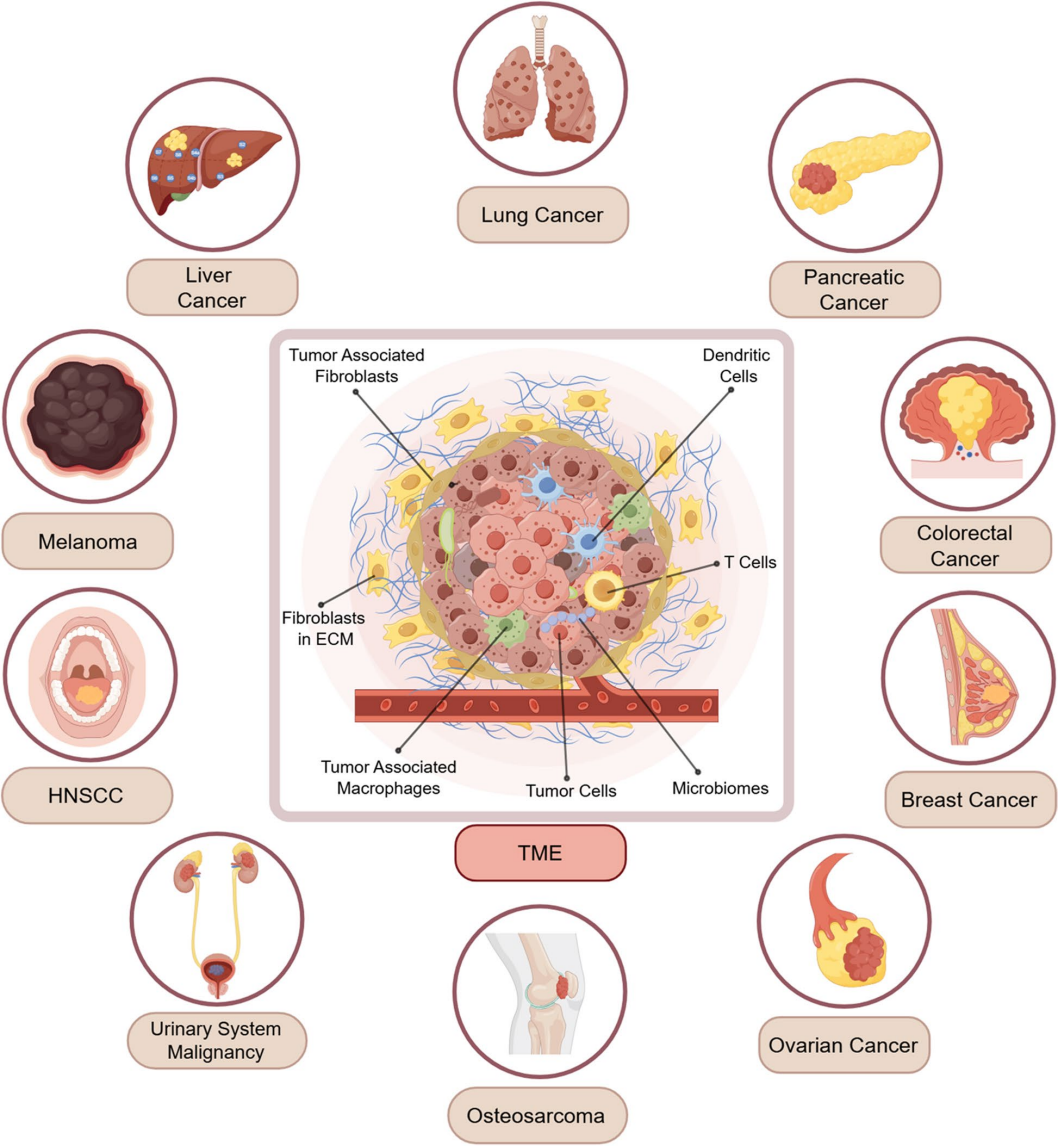
Diversity of tumor-associated microbiomes in a diverse range of malignancies. Abbreviations include HNSCC (head and neck squamous cell carcinoma) covering cancers such as oral cancer, nasopharyngeal carcinoma, and thyroid carcinoma, as well as urinary system malignancies like renal cancer, bladder cancer, and prostate cancer. (Generated using Figdraw)

Tumor-resident bacterial colonies were primarily located within both tumor cells and immune cells, with strain heterogeneity observed across different solid tumors, highlighting the type-specific characteristics of their respective microbiome compositions (Table 1, Fig. 1) [11]. Recent advances in 16S rRNA sequencing, shotgun, quantitative polymerase chain reaction (qPCR), real-time quantitative polymerase chain reaction (RT-qPCR), and metatranscriptomics have revealed that while tumor-intrinsic microbiomes generally exhibit relatively lower biomass, their influence on tumor progression remains nonnegligible [11, 23]. Additionally, tumor microbiome also exhibits the distinct microbial profiles across different malignancies, with specific bacterial communities driving tumor progression and metastasis through diverse cellular and molecular mechanisms.

**Table 1:** Studies on microbiomes diversity in a diverse range of malignancies

Malignancy Type	Tumor Microbiome Strain	General Description on Tumor Microbiome Presence as the Biomarker	Detection Technology Technologies	Ref
Colorectal Cancer	*Fusobacterium spp, E. faecalis,* ETBF, EPEC	Inflammatory response, DNA damage, and carcinogenic Wnt signaling pathway activation	RT-qPCR	[24]
Colorectal Cancer	*F. nucleatum*	Recognize Gal-GalNAc overexpressed in tumor cells via Fap2	qPCR, FISH	[25]
Colorectal Cancer	*F. nucleatum*	Activate the E-cadherin / β-catenin pathway via the FadA protein, thus leading to DNA damage and colorectal cancer cell proliferation	RT-qPCR, IHC, IF, Comet Assay, Flow Cytometry, Culture	[26]
Colorectal Cancer	*F. nucleatum*	Microbiome-derived FadA activates oncogenic and inflammatory signals by binding to E-cadherin, thereby promoting cancer occurrence and development	RT-qPCR, IHC	[27]
Colorectal Cancer	*Clostridioides difficile*	Microbiome-secreted toxin TcdB induces Wnt signaling pathway activation, ROS burst, and a tumor-promoting mucosal immune response	16S rRNA sequencing, qPCR, MALDI-TOF mass spectrometry	[28]
Colorectal Cancer	H. *pylori*	Accelerate tumorigenesis and cancer promotion through STAT3 activation, goblet cell loss, and changes in regulatory T cells and pro-inflammatory T cells	16S rRNA sequencing, scRNAseq	[29]
Colorectal Cancer	*E. coli* (CoPEC)	Creates a local high glycerophospholipid microenvironment that reduces tumor immunogenicity and decreases infiltration of CD8 + T lymphocytes	16S rRNA sequencing, spatial metabolomic profiling technologies	[30]
Colorectal Cancer	*P. anaerobius*	Promote tumorigenesis by interacting with α2/β1 integrins on CRC. The bacterial surface protein PCWBR2 binds to integrins and activates both PI3K-AKT pathway and NF-κB signaling pathways, thereby promoting cancer cell proliferation	qPCR, FISH, TEM, IHC	[31]
Colorectal Cancer	*P. gingivalis*	Induce natural killer T cells to express CHI3L1 protein, impairing anti-tumor cytotoxicity function, thereby promoting tumor immune escape and CRC progression	16S rRNA sequencing, RNA-seq, RT-qPCR, Culture	[32]
Colorectal Cancer	*S. bovis*	Recruit CD11b + TLR4 + cells to promote CRC genesis and progression	RT-qPCR, Flow Cytometry, H&E Staining, Culture	[33]
Colorectal Cancer	Sulfate-reducing bacteria	A potential environmental risk factor contributing to CRC development	16S rRNA Sequencing, RT-qPCR, IHC, Shotgun Sequencing, Culture, Nucleic Acid Hybridization	[34]
Colorectal Cancer	*H. hepaticus*	Promote the tumorigenesis in infected host colon cells	RT-qPCR, culture, IHC, Flow Cytometry	[35]
Lung Cancer	*Streptococcus and Veillonella*	Upregulate the ERK and PI3K signaling pathways enriched in lung cancer tissues	16S rRNA Sequencing, RNA-seq	[36]
Lung Cancer	*Bacteroidaceae, Lachnospiraceae, and Ruminococcaceae*	Correlate to poorer recurrence-free survival or disease-free survival by suppressing antitumor immune responses, with an increased abundance in tumor tissues	16S rRNA Sequencing	[37]
Lung Cancer	*Corynebacterium, L. iners, S. yunnanensis, P. marcusii, K. pneumonia, R. mucosa, N. macacae*	Enriched in lung cancer tissues	16S rRNA Sequencing, FISH, IHC, TEM, CLEM, Culture	[11]
Lung Cancer	*Acidovorax temporans*	Work in conjunction with smoking and TP53 gene mutation to promote lung cancer, with enrichment in smoking patients tissues with squamous cell carcinoma	16S rRNA Sequencing, RNA-seq, FISH	[38]
Lung Cancer	*P. gingivalis*	Weaken the immune response and alter the TME to promote lung cancer development and progression	IHC	[39]
Breast Cancer	*E. coli, S. epidermidis*	Promote breast cancer by inducing DNA double-strand breaks and increasing genomic instability	16S rRNA Sequencing, qPCR, IF	[40]
Breast Cancer	*M. radiotolerans*	Contribute to cancer progression with an enrichment in tumor tissues	16S rRNA Sequencing, qPCR	[41]
Breast Cancer	*F. nucleatum, Atopobium, Gluconacetobacter, Hydrogenophaga, Lactobacillus*	Enrichment in caner tissue	16S rRNA Sequencing, qPCR	[42]
Pancreatic Cancer	P. *gingivalis, Aggregatibacter actinomycetemcomitans, Alloprevotella sp.*	Associate with a significantly increased risk of pancreatic cancer	16S rRNA Sequencing	[43]
Pancreatic Cancer	*Streptococcus and Leptotrichina*	Amplify the link to cancer occurrence	16S rRNA Sequencing	[44]
Pancreatic Cancer	*Neisseria elongata and Streptococcus mitis*	Link to pancreatic cancer and chronic pancreatitis, offering a potential non-invasive biomarker source	16S rRNA Sequencing, RT-qPCR	[45]
Pancreatic Cancer	*Fusobacterium spp.*	Elevated abundance in tumor tissues	16S rRNA Sequencing	[18]
Pancreatic Cancer	*Pseudomonas, Elizabethkingia*	Suppress anti-tumor immunity with a high abundance and prevalence in tumor tissues	16S rRNA Sequencing, FISH, qPCR	[19]
Pancreatic Cancer	Malassezia spp.^*^	Promote PDAC development by activating the complement cascade response via MBL	16S rRNA Sequencing, FISH, IHC, RT-qPCR	[46]
Melanoma	*Ruminococcaceae, Peptococcaceae.g_rc4.4, Christensenellaceae, Bacteroidales.f__S24.7, Clostridiales, Erysipelotrichaceae*	The changes in the proportions of specific microbial groups can serve as potential biomarkers for tumor detection	16S rRNA Sequencing, qPCR, Culture	[47]
Melanoma	*Clostridium*	Promote tumor progression by inducing a transient permeability increase of the intestinal barrier and microbial dysbiosis, in particular proliferation of *Clostridium*-like flora and activation of stress responses associated with β-adrenergic signaling	16S rRNA Sequencing, Shotgun Sequencing	[48]
Melanoma	F. *nucleatum, S. aureus, S. capitis*	Invade tumor cells and activate tumor-infiltrating T-cell immune responses by displaying bacterial peptides via human leukocyte antigen molecules	16S rRNA Sequencing, FISH, CLEM, TEM, qPCR, Culture, Human Leukocyte Antigen Peptidomics	[49]
Melanoma	*F. nucleatum, Trueperella, Staphylococcus, Streptococcus, Bacteroides*	Enrich in tumor tissue	16S rRNA Sequencing, Metagenomics	[50]
Bone Tumor	*S. yanoikuyae, A. massiliensis, P. argentinensis*	Enrich in tumor tissue	16S rRNA Sequencing, FISH, IHC, TEM, CLEM, Culture	[11]
Bone Tumor	*Alloprevotella, Prevotella, Selenomonas*	Enrichment in tumor tissue as potential diagnostic marker for osteosarcoma	16S rRNA Sequencing, RT-qPCR	[51]
Prostate Cancer	*Propionibacterium spp., Staphylococcus spp*	The specific predominant microbiome distribution of prostate cancer tissues is associated with tumors, and specific microbial communities may become biomarkers and therapeutic targets	16S rRNA Sequencing	[52]
Prostate Cancer	S. *anginosus, A. lactolyticus* *A. obesiensis, A. schaalii, V. cambriense,* *P. lymphophilum*	Induce the chronic inflammation as the contributing factor of prostate cancer incidence	16S rRNA Sequencing, qPCR	[53]
Bladder Cancer	*F. nucleatum*	Participate in the occurrence and development of tumors by activating the β-catenin signaling pathway, inducing chronic inflammation, and inhibiting the activity of natural killer cells and T cells	16S rRNA Sequencing	[54]
Bladder Cancer	*Acinetobacter, Anaerococcus, Rubrobacter, Sphingobacterium, Atopostipes,* and *Geobacillus*	Promote tumor progression through certain metabolic pathways, which can serve as the biomarker for the risk stratification	16S rRNA Sequencing	[55]
Oral Cancer	*Escherichia, Propionibacterium, Acinetobacter, Pseudomonas*	Highly rich in tumor tissue, and *Pseudomonas* infection may impede metastasis	16S rRNA Sequencing, Shotgun, Metagenomics, Metatranscriptomics	[56]
Oral Cancer	*P. gingivalis, F. nucleatum*	Directly interact with oral epithelial cells through TLRs, stimulating tumor formation in a IL-6-STAT3-dependent manner	16S rRNA sequencing, RT-qPCR, IHC	[57]
Ovarian Cancer	*Ralstonia, Mycobacterium,* and *Variovorax sp.*	Associate with the ovarian cancer occurrence	16S rRNA Sequencing	[58]
Ovarian Cancer	*Acinetobacter*	Serve as the diagnostic biomarker of ovarian cancer	16S rDNA amplicon sequencing, Metagenomics	[59]
Glioblastoma	*Proteobacteria, Firmicutes, Actinobacteria, Bacteroidetes*	Enrichment in caner tissue	16S rRNA Sequencing, FISH, IHC, TEM, Culture, Correlative Light and Electron Microscopy	[11]

*Abbreviations*: *E. faecalis Enterococcus faecalis*, *ETBF* Enterotoxigenic *Bacteroides fragilis*, *EPEC* Enteropathogenic *Escherichia coli*, *F. nucleatum, Fusobacterium nucleatum*, *CoPEC* Colibactin-producing *E. coli*, *P. anaerobius*, *Peptostreptococcus anaerobius*, *P. gingivalis Porphyromonas gingivalis*, *S. bovis Streptococcus bovis*, *H. hepaticus Helicobacter hepaticus*, *L. iners Lactobacillus iners*, *S. yunnanensis Sphingomonas yunnanensis*, *P. marcusii Paracoccus marcusii*, *K. pneumoniae Klebsiella pneumoniae*. *R. mucosa Roseomonas mucosa*, *N. macacae Neisseria macacae*, *S. epidermidis Staphylococcus epidermidis*, *M. radiotolerans Methylobacterium radiotolerans*, *S. aureus Staphylococcus aureus*, *S. capitis Staphylococcus capitis S. yanoikuyae Sphingomonas yanoikuyae, A. massiliensis Actinomyces massiliensis*, *P. argentinensis Pseudomonas argentinensis*, *S. anginosus Streptococcus anginosus*, *A. lactolyticus Anaerococcus lactolyticus*, *A. obesiensis Anaerococcus obesiensis*, *A. schaalii Actinobaculum schaalii*, *V. cambriense Varibaculum cambriense*, P. lymphophilum Propionimicrobium lymphophilum *Malassezia spp. is a fungal genus, Wnt Wingless/Integrated, Gal-GalNAc β-D-Galactose-α-D-N-acetylgalactosamine, Fap2 *Fusobacterium* adhesin protein 2, *E-cadherin* epithelial cadherin, *FadA Fusobacterium* adhesin A, *ROS* reactive oxygen species, *STAT3* signal transducer and activator of transcription 3, *CRC* colorectal cancer, *PCWBR2* putative cell wall binding repeat 2, *PI3K* Phosphatidylinositol 3-kinase, *AKT* protein kinase B, *NF-κB* nuclear factor-κB, *CHI3L1* chitinase 3-like-1 protein, *TLR4* Toll-like receptor 4, *ERK* extracellular signal-regulated kinase, *PDAC* pancreatic ductal adenocarcinoma, *MBL* mannose-binding lectin, *TLRs* Toll-like receptors, *IL-6* interleukin-6, *IHC* immunohistochemistry, *IF* immunofluorescence, *scRNAseq* single-cell RNA sequencing, *TEM* transmission electron microscopy, *H&E Staining* hematoxylin and eosin staining; *RNA-seq* RNA sequencing, *16S rDNA* 16S ribosomal DNA

In gastrointestinal malignancies, predominant microorganisms are usually enriched in *F. nucleatum*, *Bacteroides fragilis* (*B. fragilis*)*, Prevotella, E. coli,* and *Helicobacter pylori* (*H. pylori)*, which synergistically promote carcinogenesis by activating Wnt and STAT3 pathways, causing DNA damage and inflammatory reactions [24, 28–30]. Also, the constant enrichment of *F. nucleatum* has been observed in both primary and metastatic sites, suggesting their stability and continuity during the metastatic process, along with other related microbiome (*Bacteroides, Selenomonas*, and *Prevotella* species) [21]. Lung cancer presents its own set of microbial players, such as *Streptococcus* and *Veillonella*, which further weaken the host's immune response and promote cancer progression by upregulating the ERK and PI3K signaling pathways [36]. Furthermore, *E. coli* and *S. epidermidis* induce DNA double strand breaks in breast cancer, leading to genomic instability and thereby promoting the tumorigenicity [40]. Similarly, pancreatic cancer exhibits relative abundance of *P. gingivalis* and *F. nucleatum*, which significantly elevate cancer susceptibility by inhibiting immune responses and triggering chronic inflammation [18, 43]. In the urinary system, *Akkermansia muciniphila* (*A. muciniphila*) is enriched in tumor tissue and acts as a biomarker for immunotherapy efficacy [60]. Prostate cancer is associated with *Propionibacterium spp*. and *Staphylococcus spp.*, which may serve as both biomarkers and potential therapeutic targets [52]. In bladder cancer, *F. nucleatum* promotes tumor progression by activating the β-catenin signaling pathway, inducing chronic inflammation, and suppressing immune cell activity [54]. Therefore, large-scale clinical trials can further enhance our understanding of how these interactions can alter clinical outcomes and enhance the effectiveness of immunotherapies.

### Composition of microbial species across different tumor stages

Nejman D, et al. comprehensively investigated the presence and diversity of bacterial communities, revealing how tumor microbiome influence tumor progression. The diversity of microbial species varies significantly across malignancies, underscoring that each tumor type displays unique microbiota profiles [11]. Stunningly, within solid tumors, microbiome composition may also vary across subtypes or stages even of the same malignancy type, with distinct microbial shifts being observed, suggesting that the interaction of the microbiota with the TME is critical for tumor progression.

To be more specific, a significant microbial variation was observed across different breast cancer subtypes, including estrogen receptor-positive (ER-positive), progesterone receptor-positive (PR-positive), and human epidermal growth factor receptor 2-positive (HER2-positive), with specific bacterial taxa exhibiting a varying prevalence among these subtypes [11]. For instance, triple-negative breast cancer shows a relatively simple microbiota, including *Aerococcus* and *Leishmania*. In contrast, triple-positive breast cancer exhibits a more diverse microbiota, featuring microbial communities such as *Bordetella* and *Penicillium*. Endocrine receptor-positive breast cancer displays the most complex microbiota, with species *Arcanobacterium* and *Brugia* detected. *Streptococcus* and *Balamuthia* were predominantly found in HER2-positive breast cancer. These unique microbial signatures provide new insights into the pathogenesis and progression of breast cancer [61].

Additionally, studies suggest that specific microbial communities can promote early tumor formation by activating a series of carcinogenic pathways, while the microbiome profiles may become more complex and dysregulated in advanced tumors, with the propensity of further driving tumor cell invasion and metastasis. For instance, in thyroid papillary carcinoma, Yuan L et al. revealed that the diversity of tumor microbiome in patients with advanced stages is significantly higher than that in patients with early stages [62]. Microbial diversities in stage T1/T2 were detected to be highly rich in *Pseudomonas, Rhodococcus,* and *Sphingomonas*. While those in T3/T4 exhibited greater diversity, including *Streptococcus, Granulicatella,* and *Coriobacteriales* [62]. In gastric cancer, researchers also identified shifts in the predominant microbiome community across different stages. *Romboutsia* was found to be enriched in gastric intraepithelial neoplasia, while *Kroppenstedtia*, *Parvimonas*, and *Eikenella* dominate as the disease progresses [63]. Wang et al. described microbial alterations in diversity and abundance in gastric intraepithelial neoplasia, including *Armatimonadetes*, *Chloroflexi*, *Elusimicrobia*, and *Nitrospirae.* Simultaneously, the phyla *Actinobacteria*, *Bacteroidetes*, *Firmicutes*, and *Fusobacteria* significantly witnessed a growth during the premalignant/malignant stages [64]. Similarly, researchers noted significant changes in gut microbiota abundance in CRC, with *Bacteroidetes* and *Firmicutes* diminishing while *Proteobacteria* increased as the disease progressed [65]. In fact, the significant microbiome variations are linked to specific functions, likely affecting energy metabolism critical to tumor progression, and explaining their association with advanced tumor stages [66]. Therefore, the above findings suggested that tumor microbiota exhibit specificity not only across various tumor types but also undergo alterations in composition and diversity as the disease progresses through different stages.

## Tumor microbiome and tumor initiation

### Certain microbes trigger carcinogenesis

Specific microorganisms can cause malignant transformation through a variety of mechanisms, including triggering chronic inflammation, causing DNA damage, suppressing the immune system, secreting harmful metabolites, and altering the TME [67]. These dynamic mechanisms allow microorganisms to disrupt the normal functioning of host cells and ultimately promote tumor development. Admittedly, it can be mentioned that microorganisms are not only the cause of infection, but also a contributing factor of certain type of malignancy.

**Fig. 2:**
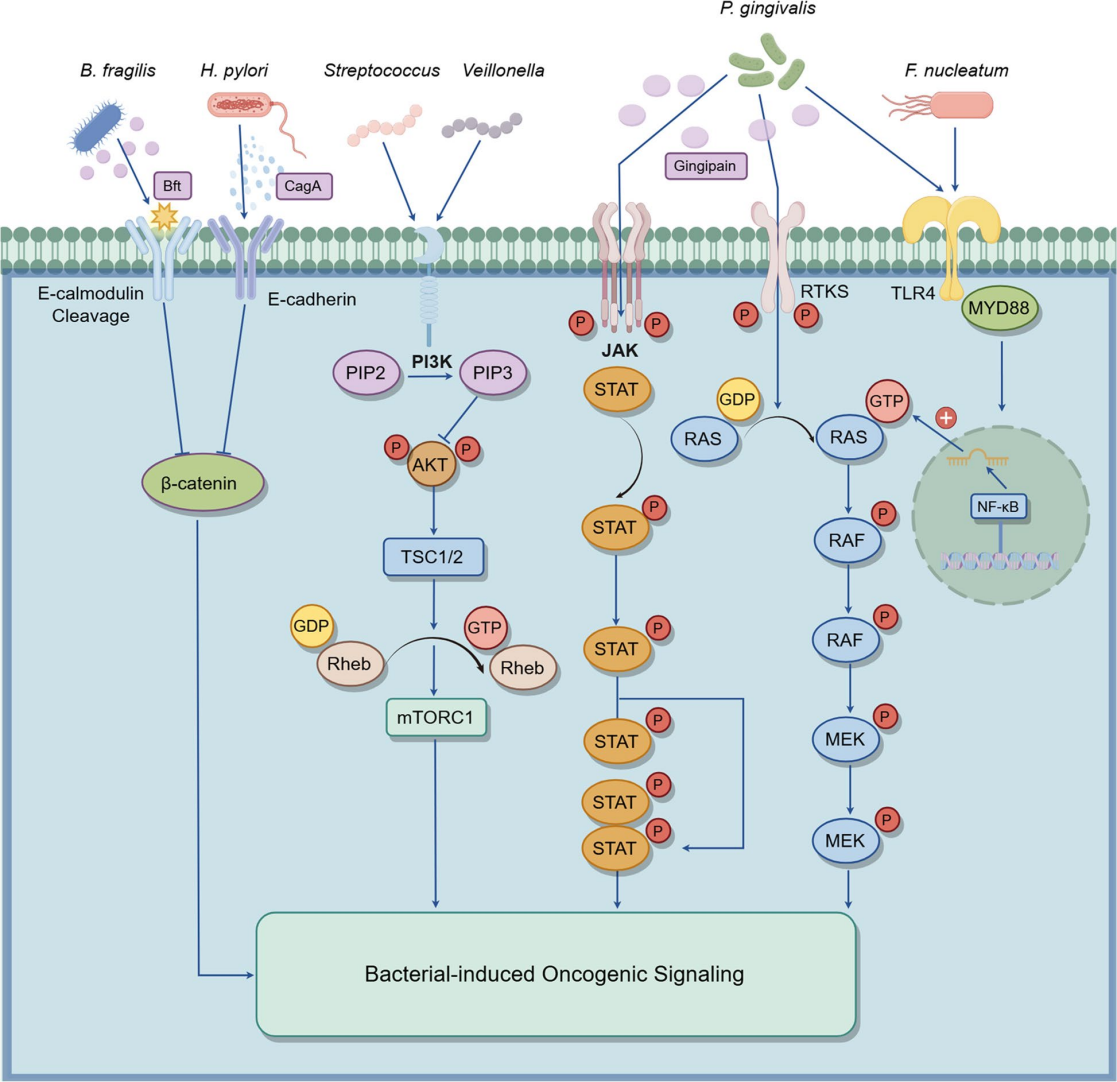
Bacterial-induced oncogenic signaling pathways activated by various microbial species. *B. fragilis* secretes *Bft*, which induces E-cadherin cleavage, while *H. pylori* injects CagA into the cytoplasm, disrupting E-cadherin and β-catenin complexes. These mechanisms result in cytoplasmic and nuclear accumulation of β-catenin. Streptococcus and Veillonella are linked to PI3K-AKT pathway upregulation. *P. gingivalis* produces Gingipain, which activates the Janus kinase-signal transducer and activator of transcription (JAK-STAT) pathway and receptor tyrosine kinases, initiating the RAS/RAF/MEK cascade and promoting cellular proliferation. *F. nucleatum* interacts with TLR4, activating NF-κB via MYD88, which contributes to inflammation and oncogenesis. (Generated using Figdraw)

To be more specific, certain microorganisms induce malignant transformation within an inseparable carcinogenic network. Firstly, chronic inflammation as one of the triggering factors exert significant influences on tumorigenesis, some microorganisms can persist in the host for a long time, thus inducing sustained inflammatory reactions. This irritable state increases the chance of cell mutations and promotes the occurrence of tumor [68]. Concurrently, bacterial toxins such as colibactin, secreted by *E. coli*, can directly cause DNA double-strand breaks, which is a crucial pathway for DNA damage. In addition, the chronic inflammation caused by microorganisms can prompt immune cells to release large amounts of ROS and reactive nitrogen species (RNS), further exacerbating DNA oxidative damage [69]. Therefore, what can be manifested in the oncogenic transformation is the participation of bacterial colonization, disrupting the cell-intrinsic homeostasis and promoting tumor incidence in an interactive manner. Besides, Microorganisms regulate host immune responses, cell proliferation, and apoptosis through various signaling pathways, such as Wnt/β-catenin and PI3K-AKT pathways, thereby promoting the initiation and progression of tumors. Exploring the interaction between tumor microorganisms and these signaling pathways can deepen our understanding of the mechanisms underlying tumor development and provide new insights for tumor prevention and treatment. These signaling pathways are illustrated in Fig. 2.

### Corresponding carcinogenic mechanisms related to microbial colonization

#### Carcinogenic pathways of certain microbiomes

##### Wnt/β-catenin Signaling

The Wnt/β-catenin signaling pathway is one representative carcinogenic pathway. Widely involved in a variety of human tumors and animal experimental tumor models, its aberrant activation is closely associated with increased tumor incidence [70]. For instance, *H. pylori* infection can contribute to gastric carcinogenesis, with some strains expressing the cytotoxin-associated gene A (CagA) protein, which they inject directly into the cytoplasm of host cells, driving gastric carcinogenesis and progression through the activation of the β-catenin signaling pathway [71]. Besides, *F. nucleatum* is featured with producing FadA that binds to E-cadherin, which enhances the production of pro-inflammatory cytokines and transcription factors [72, 73]. Another route that activates β-catenin pathway is *Bacteroides fragilis* toxin (*Bft*) produced by *B. fragilis*, which stimulates cleavage of E-cadherin and contributes the occurrence of neoplastic conditions [74]. Moreover, some of the viruses also mediate the activation of this oncogenic pathway. For instance, HBx protein of Hepatitis B virus (HBV) can activate Wnt/β-catenin as the hepatocarcinogenesis stimulus [75].

##### PI3K-AKT Signaling

The PI3K pathway is aberrantly activated in multiple malignancies, which accelerates the growth and spread of tumor cells by enhancing cell proliferation, promoting cell survival, and inhibiting apoptosis [76]. For instance, it has been found that certain oral commensal bacteria (*Streptococcus* and *Veillonella*) are closely associated with the upregulation of the PI3K signaling pathway in the lower respiratory tract of lung cancer patients. In vitro experiments also verified this finding, further suggesting a potential pathogenic mechanism between microorganisms and carcinogenesis [36]. Gustafson et al. also confirmed this assumption, showing that the PI3K signaling pathway was significantly activated in their tracheal epithelial cells in smokers with lung cancer or precancerous lesions [77].

##### ERK signaling

The ERK signaling pathway plays a key role in tumor occurrence by promoting cell proliferation as well as anti-apoptosis functions. The pathway belongs to the Rat sarcoma virus (Ras)-Rapidly accelerated fibrosarcoma (Raf)-Mitogen-activated protein kinase kinase (MEK)-ERK signaling cascade, which is often initiated by the activation of growth factors or cytokines, and ultimately promotes the expression of genes associated with tumor cell proliferation and growth by phosphorylating target proteins and transcription factors [78]. Moreover, microbiome-induced carcinogenesis is also correlated with this ubiquitous pathway, *F. nucleatum* infection was found to promote the proliferation and invasive ability of CRCs, by activating TLR4 signaling pathway and acting on myeloid differentiation primary response gene 88 (MYD88), which in turn activated NF-κB, led to upregulation of microRNA 21 (miR21) expression. Then the miR21 overexpression promoted tumor progression by inhibiting RAS GTPase (RASA1) and ultimately upregulating the ERK pathway [79]. Likewise, *F. nucleatum* could also enhance tumor cell proliferation, migration, and invasion, contributing to metastasis through activating the TLR4/MYD88 signaling pathway [80]. Additionally, Mu et al. proposed the role of *P. gingivalis* in CRC. It was found that *P. gingivalis* was able to invade colorectal cancer through its key virulence factor *gingipain*, a cysteine protease. Upon successful invasion, they can significantly promote the cellular proliferation by activating mitogen-activated protein kinase (MAPK)/ERK signaling pathway [81].

##### JAK-STAT signaling

The JAK-STAT signaling pathway plays a pivotal role in cytokine-driven signaling and cell cycle regulation [82]. Research indicates that certain microbiomes can influence host cell apoptosis and proliferation by modulating this pathway. For example, *P. gingivalis* has been shown to interfere with the host cell's mitochondrial apoptotic pathway by manipulating JAK-STAT signaling, thereby inhibiting programmed cell death. This modulation extends host cell survival and enhances cell proliferation, suggesting a potential oncogenic role for *P. gingivalis* in tumorigenesis and highlighting the intricate interplay between microbial activity and host cellular pathways [83].

##### Other signaling

In fungal communities, studies have also shown that fungal dysbiosis, particularly the enrichment of fungi of the genus *Malassezia*, is strongly associated with the development of PDAC. Fungi recognize cell wall glycoconjugates via MBL, which activates the complement cascade reaction and promotes tumor growth. Removal of the fungal population or deletion of MBL and complement component 3 (C3 complement) inhibited tumor progression, suggesting that MBL-driven complement activation plays a key role in PDAC carcinogenesis [46]. Apart from complement system, Fu A et al. revealed that bacteria-induced inhibition of Ras homolog family member A (RhoA)/Rho-associated coiled-coil containing protein kinase (ROCK) signaling pathway could increase the defensive capacity against fluid shear stress, thereby promoting metastasis of breast cancer cells [22]. Also, following a multi-omics analysis of triple-negative breast cancer (TNBC) patients, it was found that *Clostridiales* bacteria and their metabolites trimethylamine N-oxide (TMAO) could induce protein kinase-like endoplasmic reticulum kinase (PERK), thus increasing anti-tumor immunity [84].

#### DNA damage

Some microorganisms, especially bacterial communities in the gastrointestinal tract, possess the potential of directly causing DNA damage and leading to genome mutation. A representative one is *E.coli*. Research shows that *E.coli* carries polyketide synthase (Pks) pathogenicity islands that encode enzymes that synthesize colibactin, which is thought to alkylate adenine in DNA and induce double-strand breaks [85]. It has also been found that the colonic mucosa of patients with familial adenomatous polyposis (FAP) is enriched with biofilms composed of *E.coli* and *B. fragilis* carrying the oncotoxin genes for secreting colibactin and *Bft*. Both toxins create opportunities for pathogens to invade the mucus layer of the colon and colonize the polyp, with interleukin-17 secretion leading to DNA damage, and thus accelerating the early onset and progression of the tumor [86]. This study initiated research into microbiome-derived toxins and their impact on DNA damage, Wilson MR et al. revealed colibactin as a small-molecule genotoxin, which could alkylate DNA via an uncommon cyclopropane-based electrophilic compound presumably derived from an unstable colibactin-DNA adduct [87].

Besides, the toxin *Bft* produced by ETBF is another oncogenic factor contributing to neoplastic transformation, mainly upregulating spermine oxidase (SMO) expression in colonic epithelial cells, leading to the production of ROS and eventually triggering DNA damage [88]. Moreover, in oral cancer, *P. gingivalis* and *F. nucleatum* promote carcinogenesis through host genome alterations, triggering the production of inflammatory cytokines, cell proliferation, inhibition of apoptosis, invasion and migration [89]. In breast cancer, patients exhibit different bacterial communities, with higher abundance of *Bacillus*, *Enterobacteriaceae*, and *Staphylococcus* compared to healthy women, and these bacteria are capable of triggering DNA double-strand breaks in vitro [40]. Additionally, secreting membrance vesicles as an effective method can also contribute to the occurrence of neoplasm. *Actinomyces odontolyticus*-secreted vesicles containing lipophosphatidic acid can induce NF-κB signaling and generate excessive ROS in colonic epithelial cells, promoting inflammatory signaling through the Toll-like receptor 2 (TLR2) pathway and co-localizing with mitochondria in colonic epithelial cells, leading to mitochondrial dysfunction, excess ROS production, and DNA damage [90].

Apart from bacterial communities, oncoviruses are another type of carcinogenic microorganisms due to their ability to incorporate pathogenic genomes into the host chromosome, as seen with HBV [91]. Considering that genomic instability is a key feature of tumor development, a decisive factor contributing to tumorigenesis is the DNA damage response (DDR) system, which plays a crucial role in maintaining genomic stability [92]. Abnormalities in the DDR system, which compromise DNA repair mechanisms, have been widely identified in numerous genes across various types of tumor, contributing significantly to the processes that drive tumor initiation and progression [92, 93]. However, excessive DDR activation can also contribute to tumorigenesis. HPV E7 protein supports viral DNA amplification by inducing differentiated cells to re-enter S phase and activating the DDR system [94]. Rapid replication of the HPV double-stranded circular genome creates aberrant DNA structures that in turn attract and activate DDR proteins, indicating that HPV relies on DDR activation to complete its life cycle [95]. Of note, the descriptions above further strengthen the understanding of how certain microorganisms may induce tumor incidence through DNA mutations. However, whether DNA damage alone is sufficient to form mutations that lead to malignancy remains questionable, and this issue requires further in-depth studies.

#### Epigenetic modification

Epigenetic modifications are a form of chemical modifications, such as DNA methylation and histone modifications, that do not alter the DNA sequence but are capable of modulating gene expression and affecting cellular function by regulating genome activity [96]. Microorganisms can promote carcinogenesis and progression through specific epigenetic modification mechanisms, as evidenced by triggering aberrant host DNA methylation, altering histone modification status, and regulating the expression of non-coding RNAs. These mechanisms allow bacteria to disrupt normal cellular regulation by affecting gene expression in host cells, ultimately inducing tumor formation.

For instance, studies have shown that *F. nucleatum* is associated with long interspersed element-1 (LINE-1) hypomethylation in esophageal cancer, influencing overall genomic methylation levels. However, the effect of *F. nucleatum* on clinical outcomes was not affected by LINE-1 hypomethylation. This suggests that *F. nucleatum* may influence tumor development by altering DNA methylation [97]. Another study demonstrated that *B. fragilis, E. coli, F. nucleatum,* and *K. pneumoniae* were significantly enriched in CpG island methylator phenotype-positive (CIMP-positive) cases and were associated with methylation of the MutL homolog 1 gene after analyzing 203 CRC patients samples, indicating that specific bacteria may drive CRC progression by altering CpG island methylation [98]. Moreover, Qiao et al. found that *Mycoplasma hyorhinis* (*M. hyorhinis*) promotes tumor progression by enhancing nuclear polyploidy in hepatocellular carcinoma (HCC). *M. hyorhinis* in the intestine can infect the liver retrogradely, which degrades mitochondrial fusion protein 1 mRNA in an N6-methyladenosine (m6A)-dependent manner, leading to mitochondrial fragmentation and increased tumor stemness [99]. *H. pylori* significantly increased the methylation levels of several CpG islands in healthy individuals. In *H. pylori*-negative individuals, the methylation levels of specific CpG islands were associated with gastric cancer risk, whereas in *H. pylori*-positive individuals, the changes in methylation levels were more diverse, and methylation of some regions was associated with gastric cancer [100]. Additionally, a retrospective study suggested that *F. nucleatum* could induce m6A methylation to promote metastasis in esophageal squamous carcinoma through a methyltransferase-like 3 (METTL3)/YTH domain family protein 1 (YTHDF1)/myelocytomatosis oncogene (c-Myc) axis, after extracting and analyzing tumor tissues from 22 patients [101]. The detection of m6A sequencing in infected Eca109 cells saw its peak elevation, and METTL3 was found in m6A-targeted regions. Consequently, methylation-induced METTL3 activation upregulated c-Myc protein expression, promoting cellular migration and proliferation [101]. In lung cancer, butyrate-producing microbial communities were found to upregulate genes related to H19, a long non-coding RNA (lncRNA), by inhibiting histone deacetylase 2 (HDAC2), thereby promoting histone H3 lysine 27 acetylation (H3K27 acetylation) and enhancing H19 promoter activity [102].

#### Chronic inflammation

Chronic inflammation has been widely recognized as a driver of carcinogenesis, particularly in gastrointestinal malignancies [103]. The NF-κB signaling pathway, as one of the most widely distributed pathways contributing to inflammation, participates in multiple biological activities TLRs are crucial components of the innate immune system, which activate NF-κB inflammatory pathways by recognizing pathogen-associated molecular patterns (PAMPs) and endogenous molecules, thus leading to an increase in pro-inflammatory cytokines and chemokines [104]. As essential nodes connecting inflammation to carcinogenesis, TLRs play a critical role. For example, *F. nucleatum* infection promotes CRC initiation by activating the TLR4-MYD88-NF-κB signaling pathway, upregulating miR21 expression, and inhibiting the level of RAS GTPase RASA1 [79]. Concurrently, cytokines from the interleukin (IL) family are essential to inflammation occurrence and progression. For instance, elevated levels of IL-17, IL-21, and IL-22 were found in *F. nucleatum*-infected mice [79]. In addition to creating a pro-inflammatory microenvironment through secreting IL-6, IL-8, and tumor necrosis factor-alpha (TNF-α), *F. nucleatum* also amplified immunosuppressive cells to decrease anti-tumor immunity, such as myeloid-derived suppressor cells (MDSCs) and tumor-associated macrophages (TAMs), disrupting the intestinal epithelial barrier and further exacerbating inflammation and tumor progression [105].

**Fig. 3:**
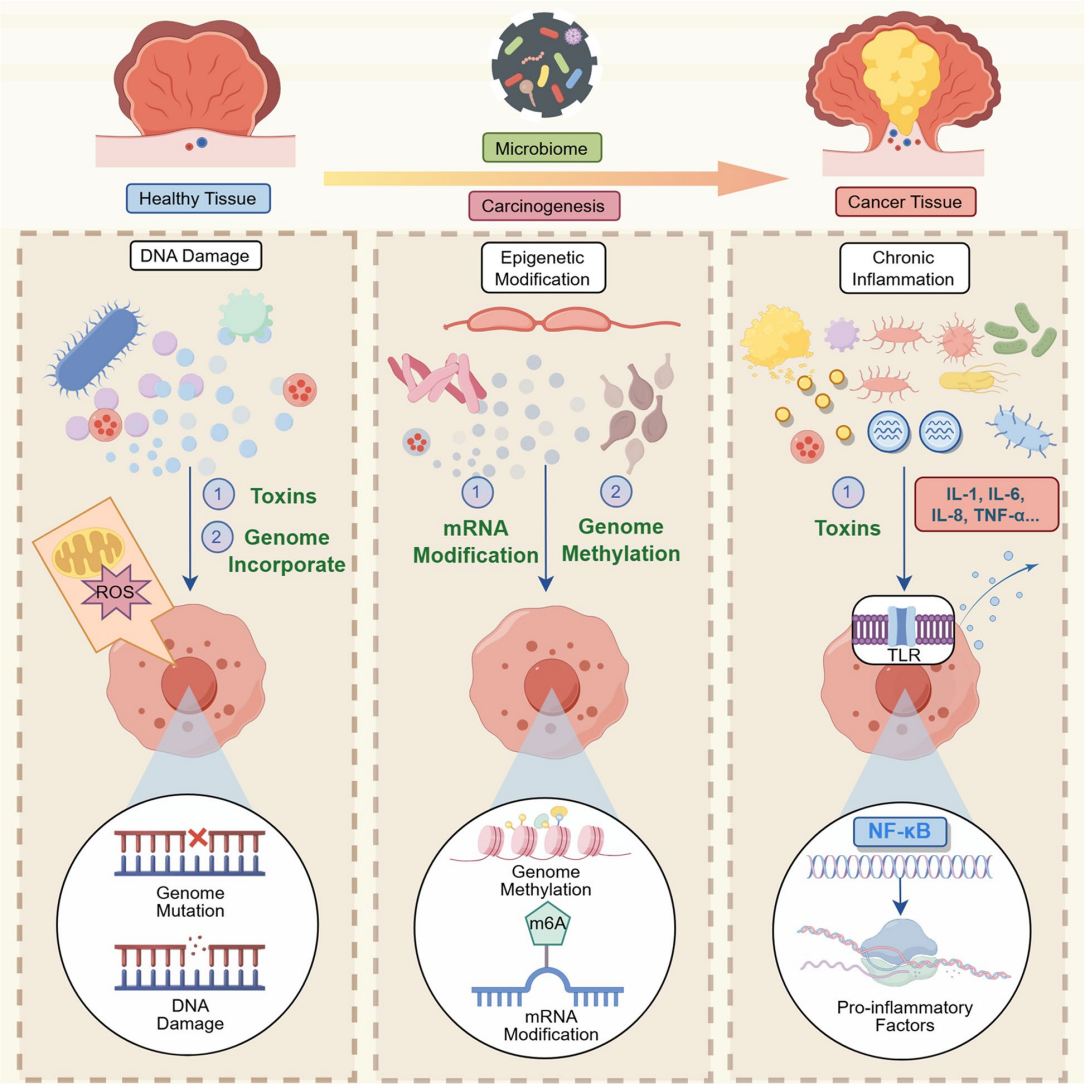
Carcinogenic mechanisms associated with microbial colonization, including DNA damage, epigenetic modification, and chronic inflammation. Microbiome-derived toxins cause DNA damage by generating ROS and induce genome mutations through incorporation of oncogenes into host DNA. Carcinogenic microbiomes affect cancer incidence by altering mRNA expression and modifying genome methylation levels. Chronic inflammation arises from a variety of pro-inflammatory factors, such as IL-1/6/8 and TNF-α, where bacterial toxins bind to TLRs, activating NF-κB and other inflammatory pathways. These processes facilitate the transformation from healthy to cancerous tissue. (Generated using Figdraw)

Besides *F. nucleatum*, *B. fragilis* has also been shown to trigger inflammatory responses, ETBF triggers CRC through secreting *Bft*, which activates β-catenin protein and NF-κB signaling pathways through cleavage of E-cadherin and contributes to the secretion of pro-inflammatory factors, such as IL-8. In addition, *Bft* independently promotes inflammation and cell proliferation through the MAPK pathway, and these signaling pathways work together to contribute to colon carcinogenesis and progression due to ETBF infection [106]. In oral cancer, *P. gingivalis* triggers inflammation and promotes carcinogenesis through several mechanisms. First, it contributes to the upregulation of pro-inflammatory factors such as IL-1, IL-6, IL-8, and TNF-α through activation of TLR4, it also inhibits apoptosis and enhances epithelial cell survival by activating the JAK1/STAT3 and PI3K/AKT signaling pathways. In addition, it promotes epithelial-mesenchymal transition (EMT) by up-regulating matrix metalloproteinases 9 and matrix metalloproteinases 13, suppressing T cell function and promoting tumor immune escape [89]. Moreover, *P. gingivalis* and *F. nucleatum* have been shown to activate the IL-6/STAT3 axis and induce chronic inflammation, thereby promoting the occurrence of oral squamous cell carcinoma. This inflammation leads to upregulation of pro-inflammatory cytokines and signaling pathways, these bacteria interact directly with oral epithelial cells through TLRs, promoting cell proliferation and enhancing the expression of tumor related molecules, thereby driving the development and progression of tumor [57]. Therefore, microorganisms drive tumor development and progression by inducing chronic inflammation, promoting the release of pro-inflammatory factors, and creating an inflammatory immune microenvironment. The carcinogenic mechanisms associated with microbial colonization, including DNA damage, epigenetic modification, and chronic inflammation, are illustrated in Fig. 3.

### Representative microorganisms associated with carcinogenisis

Currently, the most recognized carcinogenic microbiota include a wide range of bacteria, fungi, and viruses [107]. They exhibit complex functions that increase host susceptibility to tumor. For instance, *H. pylori* causes persistent inflammation, destroys the gastric mucosa, leading to recurrent cellular damage and increasing the risk of DNA mutation. Additionally, *H. pylori* disrupts the host immune response through the production of toxins such as CagA and vacuolating cytotoxin A, enabling tumor cells to escape immune surveillance as well as promoting aberrant cell proliferation and transformation through activation of multiple oncogenic signaling pathways [108]. The most recognized microbial community contributing to CRC carcinogenesis is *F. nucleatum*, which modulates host immunity and selectively amplify tumor-infiltrating myeloid cells, especially MDSCs and TAMs. Another function includes upregulating pro-inflammatory genes cyclooxygenase-2 (COX-2) and IL-6, creating a tumor-suppressive and pro-inflammatory microenvironment [105].

Human papillomavirus (HPV) of high-risk types such as HPV16 and HPV18, causes cervical cancer via the E6 and E7 proteins. E6 protein binds to the tumor suppressor gene p53, leading to its degradation, weakening host DNA repair ability, and increasing genomic instability, while E7 protein attaches to retinoblastoma protein (Rb) and promotes uncontrolled cell cycle proliferation. This cooperative action leads to the tumorsuppressive gene compromise, thus contributing to cellular immortalization [109]. Hepatitis B and C viruses can cause liver cancer through long-term hepatitis and genetic damage to liver cells. HBV can integrate into sarco/endoplasmic reticulum calcium ATPase 1 (SERCA1) gene, disrupting calcium homeostasis [91]. HBx of HBV, a multifunctional regulatory protein, can inhibit the tumor suppressor gene p53 and activate pro-cancer pathways such as Wnt/β-catenin, acting as a stimulus for hepatocarcinogenesis [75]. Additionally, hepatitis C virus (HCV) promotes the development of hepatocellular carcinoma through a long-term chronic inflammation, repeated damage and repair ultimately resulting in secondary liver fibrosis, increasing genomic instability. Ubiquitously, HCV protein also promotes cell proliferation and inhibits apoptosis by activating oncogenic signaling pathways such as NF-κB and MAPK, accelerating the progression of tumor [110]. EB virus (EBV) possesses the propensity to activate multiple oncogenic pathways related to nasopharyngeal carcinoma, including PI3K-AKT, mechanistic target of rapamycin (mTOR), NF-κB, and JAK-STAT, through its encoded proteins latent membrane protein 1 (LMP1), latent membrane protein 2A (LMP2A), Epstein-Barr nuclear antigen 1 (EBNA1), as well as non-coding RNA such as BamHI A rightward transcript microRNA (BART miRNA). These viral-derived proteins promote carcinogenesis by promoting cell proliferation, inhibiting cell apoptosis, inducing EMT, and immune escape [111].

## Microbiome-mediated tumor progression

### Role of tumor microbiome in biological process

**Fig. 4:**
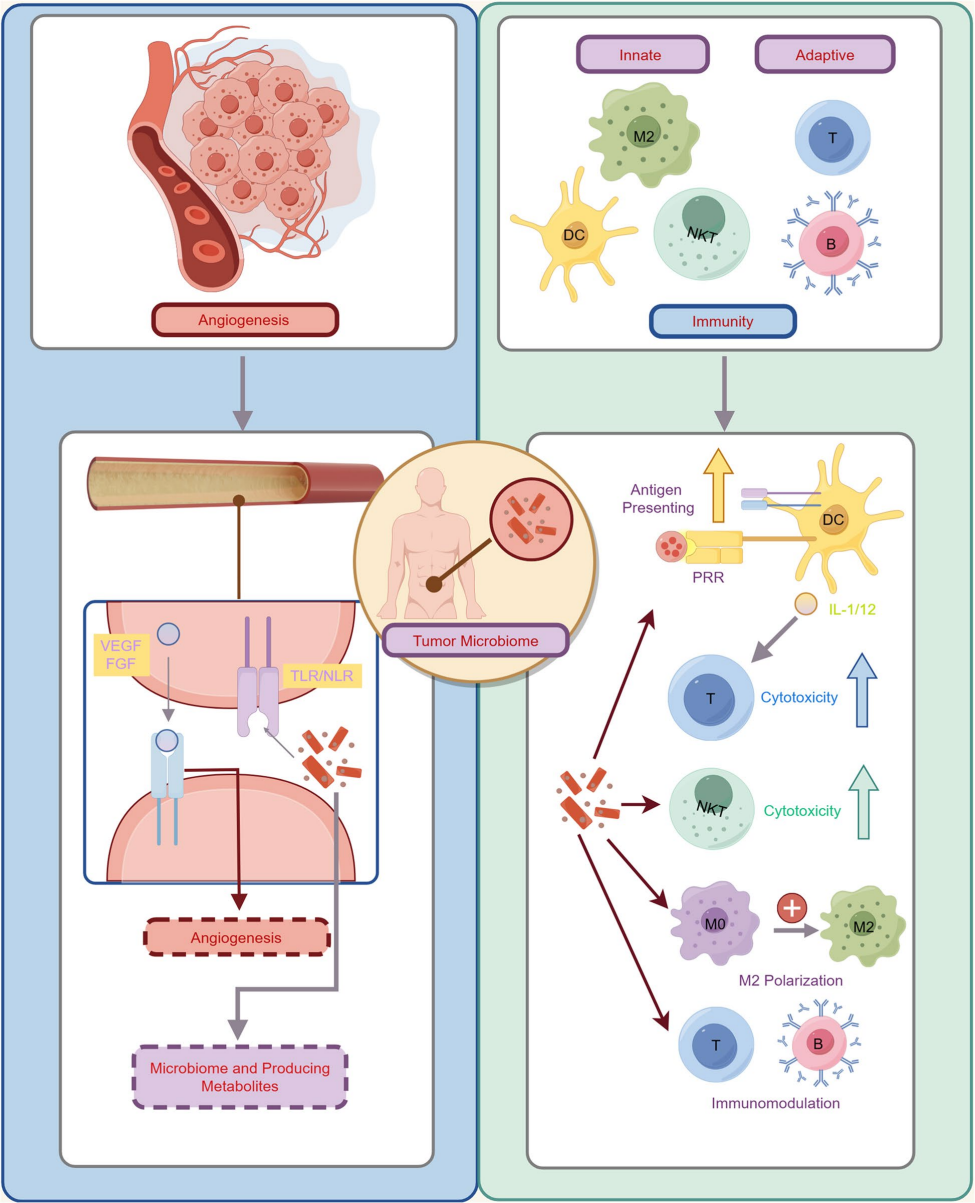
Role of the tumor-associated microbiome in promoting angiogenesis and modulating immunity during tumor progression. In angiogenesis, microbial metabolites activate endothelial cells through TLRs and NLRs, stimulating VEGF and FGF release to promote blood vessel formation. For immunity, microbial components bind to PRRs, enhancing antigen presentation and inducing IL-1/12 secretion to boost cytotoxic T-cell activity. Additionally, these interactions can increase the cytotoxic effects of NKT cells and promote M2 macrophage polarization, modulating the adaptive immune response. (Generated using Figdraw)

Microorganisms function not only as important regulators of host physiological activities, but also induce tumor onset, progression, metastasis, and modulate therapeutic efficacy. By triggering inflammation, DNA damage, regulating metabolism, and modulating immunity, the microbiome can create a favorable microenvironment for tumor cells to survive and proliferate [112]. Additionally, specific microbial communities can support tumor spread by influencing angiogenesis and tumor immunity (Fig. 4). Therefore, angiogenesis, as one of the indispensable conditions for tumor growth, is a key mechanism through which microorganisms influence the biological activities of neoplastic cells [113].

To be more specific, microbiota-induced angiogenesis has been explored in detail. Firstly, microbiome-derived metabolites such as short-chain fatty acids (SCFAs) and hydrosulfides can activate signaling pathways such as vascular endothelial growth factor (VEGF) in host cells [114, 115]. And quorum sensing peptides secreted by pathogenic bacterial communities such as *E. coli* have been found to enhance tumor angiogenesis by upregulating the expression of VEGF, thereby providing nutrients and oxygen to breast cancer cells and promoting tumor growth and metastasis [116]. Besides, microbiome-related angiogenesis sometimes can also indirectly modulate immune responses, certain pathogens can activate endothelial cells by regulating TLRs or NOD-like receptors (NLRs), leading to cell proliferation and neointimal formation [117]. Both TLRs and NLRs can recognize bacterial components, which trigger the production of pro-inflammatory cytokines, such as IL-6 and IL-8, through the NF-κB pathway, mediating mucosal angiogenesis [118]. Of note, the microbiota itself acts as an antigenic stimulus capable of promoting the release of angiogenic factors such as VEGF, fibroblast growth factor (FGF) and other angiogenic factors. Therefore, after affecting the host tumor angiogenesis, setting a solid foundation for shaping the TME, microorganisms can also degrade the extracellular matrix (ECM) by upregulating the activity of matrix metalloproteinases (MMPs), thereby promoting tumor vascularization and tumor cell infiltration. For instance, *H. pylori* promotes basement membrane degradation in gastric tissues by significantly increasing MMP-2 and MMP-9 activity by threefold and 19-fold respectively, and an increase in tissue macrophages acted in conjunction with MMP-9 [119].

Additionally, the propensity of microbiomes regulating the immunity has been studied for decades, it is well elucidated that manipulating the microbiomes can exert innumerable influences on tumor-related immune system, including both innate and adaptive. From the perspective of innate immunity, DCs take on antigen-presenting duties through specific pattern recognition receptors (PRRs). Through TLR4 signaling, microbial community translocation is likely to enhance CD8 + T cell activation [120]. In the gut, tissue-resident CD103 + DCs increase IL-1 and IL-12 secretion when exposed to commensal bacterial communities, which is critical for attracting activated cytotoxic T-cell responses as immunomodulatory effects of chemotherapy and immunotherapy [121]. Moreover, increasing studies indicate unique antigen-stimulating characteristics of microbes, one of which is the tumor neoantigen (NeoAg) mimicry [122]. It has been found that clonally expressed NeoAg can be acquired by cross-presenting DCs and present multiple NeoAg simultaneously. This dual NeoAg presentation is associated with a mature phenotype and greater stimulatory capacity of DCs. In this way, DCs are able to enhance interactions with T cells and induce more effective anti-tumor responses. Thus, clonal NeoAg expression helps DCs improve their antigen-presenting function and promotes stronger anti-tumor T-cell responses. Given this assumption, microbiome-based NeoAg mimicry will also boost tumor presenting efficacy. Natural killer T cells (NKT) cells, known for their cytotoxic tumor-eliminating activity, have an interactive relationship with C-X-C chemokine receptor 6 positive (CXCR6 +) gut microbiome. The activation of NKT cell enrichment depends on the bile duct conversion in a C-X-C motif chemokine ligand 16 (CXCL16) manner, so the aberrant microbiome translocation in the gut will diminish NKT cell accumulation, thus impeding anti-tumor effects [123]. Regarding TAMs, the subpopulations of macrophages (M0, M1, and M2) coexist in human tissues with dynamic transforming characteristics. Being endowed with immaturity, the M0 phenotype has the potential to transform into other subpopulations to gain functions according to immediate circumstances. M1 and M2 exhibit the antagonistic functions, the former is perceived as a pro-inflammatory initiator, while the latter is considered anti-inflammatory. Moreover, M1 can suppress the growth of microbial communities and tumor tissues while M2 is likely to render the opposite, revealing the possibility of managing the balances of the 3 subpopulations [124]. M2-polarized macrophages can suppress antitumor immunity, enhance tumor drug resistance, and promote tumor invasiveness by secreting cytokines and chemokines [125, 126]. For instance, in desmoplasia-resistant prostate cancer, Cathepsin K (CTSK) accelerates tumor growth and metastasis by promoting the polarization of M2-type TAMs and modulating the TME [127]. However, whether TAMs can subsequently interact with cancer cells in a specific TME remains uncertain [128, 129]. In terms of the adaptive immunity, Sethi V et al. proposed that the gut microbiome modulates tumor growth, once depleted, it contributes to an increase in interferon-gamma (IFN-γ) with a corresponding decrease in IL-10/17 derived from CD8 + T cells, suggesting that the gut microbiome community requires adaptive immunity to exert its immunomodulatory effects [130]. The interplay between microbial communities and adaptive immunity has initiated the possibility of its use in immunotherapeutic modulation, evidenced by the finding that the composition of microbial communities significantly influences patients' clinical responses to anti-programmed death-ligand 1 (anti-PD-L1) therapy. The most recognized gut microbiome favorable to ICIs efficacy include *Bifidobacterium* and *A. muciniphila*, whose purine metabolite Inosine has been explored to induce TME reprogramming and improve immunotherapy [131].

### How tumor microbes drive tumor growth

**Fig. 5:**
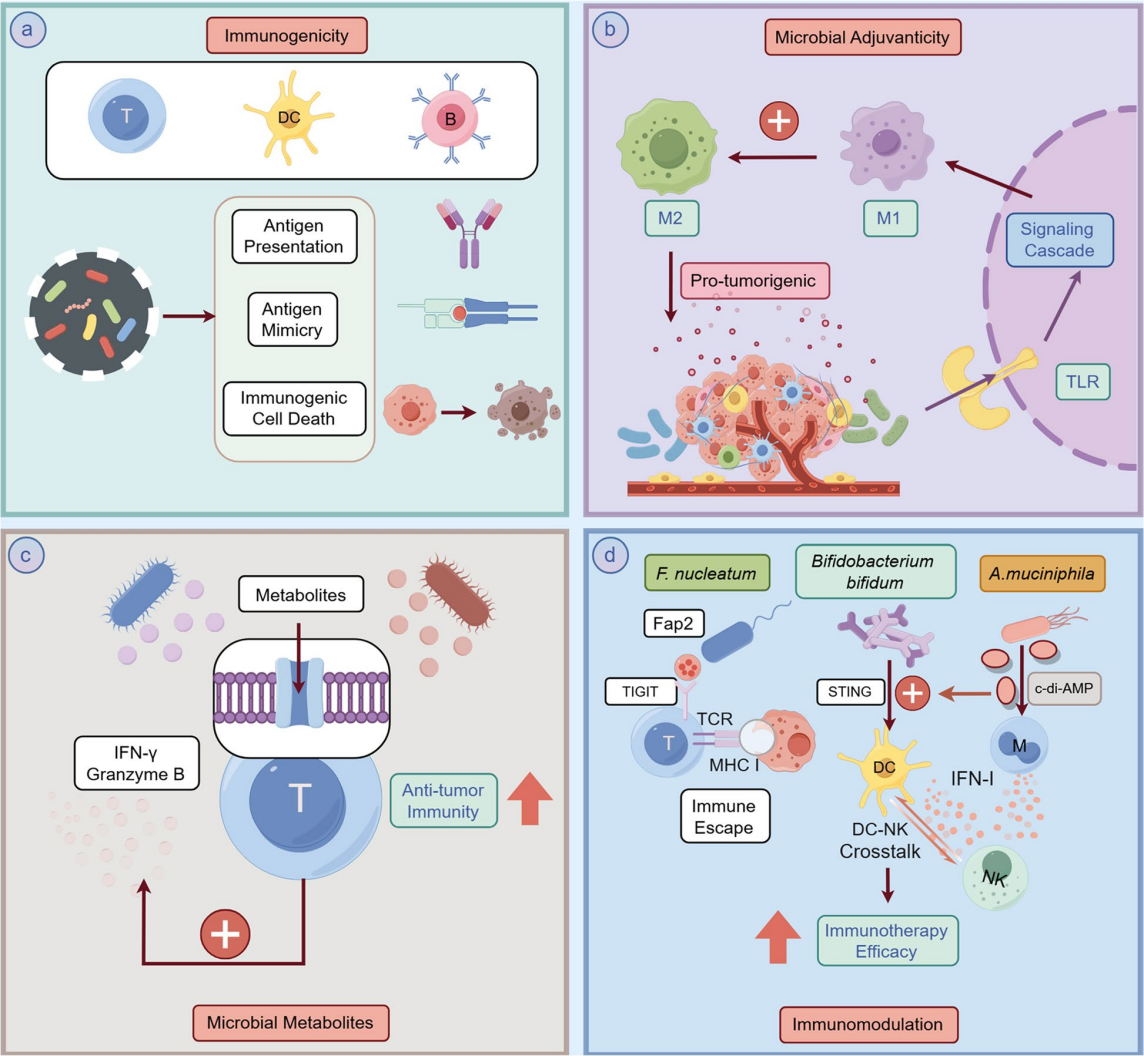
Overview of tumor microbiome's influences on the tumor immune microenvironment. **a** microbial components enhance tumor immunogenicity through antigen presentation, mimicry, and immunogenic cell death; **b** microbial signals activate immune cells, such as M1 and M2 macrophages, via TLR signaling, modulating the immune response; **c** microbial metabolites further stimulate anti-tumor immunity by boosting T-cell responses, including IFN-γ and Granzyme B production; **d** microbiome-based immunomodulation, *F. nucleatum* promotes immune evasion by interacting with TIGIT on T cells, inhibiting tumor-killing activity; *Bifidobacterium bifidum* enhances immunotherapy by activating STING and interferon pathways; *A. muciniphila*–derived c-di-AMP, as a STING agonist, boosts anti-tumor immunity by inducing IFN-I release from macrophages and enhancing NK-DC interactions. (Generated using Figdraw)

Mechanistically, tumor microbiome contributes to tumor progression and growth mainly through the immune landscape, they dynamically modulate the tumor immune microenvironment together with specific immune cells, which involves cytotoxic T cell activation through antigen presentation and antigen mimicry, inducing immunogenic cell death to release tumor antigens, modulation of immune responses via pattern recognition receptors (TLRs and NLRs), microbial metabolites affecting immune cell activity, and inhibition of immune cell function through interaction with immune checkpoint molecules [132]. The general mechanisms are illustrated in Fig. 5. To be more specific, the tumor immune landscape involves the immunogenicity and immunomodulation. The former involves increasing the immunogenicity of tumor cells, while the latter reinforces the immunomodulatory effects, such as microbial adjuvanticity, microbiome-derived metabolites, and immune checkpoint inactivation, ultimately shaping the TME to either support or suppress tumor progression. For instance, molecular mimicry of tumor antigens that share homology with microbial epitopes provides cross-reactive T cells with optimal targets for recognition and attack, such as *Enterococcal Prophage* [133]. Another representative of the tumor microbiome community is the oncolytic virus (OVs), which can enhance anti-tumor immune responses by selectively infecting and replicating tumor cells, leading to tumor cell lysis and inducing immunogenic cell death (ICD). They release viral particles to reinfect neighboring tumor cells while releasing tumor-associated antigens, danger-associated molecular patterns (DAMPs), and cytokines, which in turn recruit and activate DCs, NKs, and T cells to comprehensively enhance anti-tumor immunity [134].

In the context of microbiome-based immunomodulation, in a mouse pancreatic cancer model, tumor microbes can induce differentiation of M2-type TAMs by selectively activating TLRs in monocytes. Removal of microbes with antibiotics significantly enhanced T cell activation, promoted differentiation of M1-type TAMs, and upregulated programmed death-1 (PD-1) expression, while reducing MDSCs and M2-type TAMs within the tumor tissues [19]. Besides, microbiome-derived metabolic substances, such as SCFAs that are produced by intestinal anaerobes fermenting dietary fibers and consist mainly of acetic, propionic and butyric acids, possess the trait of suppressing tumor-associated inflammation and maintaining intestinal health. Butyric acid elevates IL-10 and retinoic acid levels in the intestinal microenvironment by binding to G-protein-coupled receptor 109A (GPR109A) on the surface of immune cells, thereby promoting the differentiation of initial T cells to regulatory T cells and facilitating their proliferation, this process effectively suppresses the pro-tumor inflammatory response [135]. Apart from that, butyric acid enhances the immune system's attack on tumor cells by inhibiting histone deacetylases (HDACs) and promoting the expression of effector molecules, such as IFN-γ and granzyme B, in CD8 + cytotoxic T lymphocytes (CTLs). Besides, butyric acid induces the conversion of IL-17-secreting CD8 + T (Tc17) cells to CTLs, further enhancing the anti-tumor immune response [136]. However, *Roseburia*-derived butyrate at low concentration was also revealed to promote polarization of M2 macrophages through inhibiting HDAC2 and enhancing H19 gene expression, thus enhancing tumor metastasis [102].

Beyond that, the metabolite TMAO from the genus *Clostridiales* is more abundant in immunologically active tumors, and patients with higher levels of TMAO responded better to immunotherapy. Mechanistic studies suggest that TMAO induces tumor cells to undergo pyroptosis by activating the endoplasmic reticulum stress kinase PERK, which in turn enhances CD8 + T cell-mediated anti-tumor immune responses [84]. Concurrently, microbial stimulation of inhibitory checkpoints regulates tumor immune microenvironment. Research has revealed that *F. nucleatum*, present in the TME, promotes tumor cells immune escape, they can use Fap2 protein to directly interact with T cell immunoglobulin and ITIM domain (TIGIT) expressed on NK cells and T cells to inhibit the tumor-killing activity. This phenomenon revealed a bacterial-dependent immune escape mechanism in which tumors evaded immune surveillance by inhibiting immune cell function [137]. Shi et al. found that *Bifidobacterium bifidum* enhances anti-cluster of differentiation 47 (anti-CD47) immunotherapy by activating the stimulator of interferon genes (STING) and interferon signaling pathways [138]. CD47 is a widely expressed cell receptor that regulates macrophage phagocytosis, neutrophil transmembrane migration, and the activation of immune cells. Numerous studies have shown that various types of malignancies evade immune system attacks by overexpressing CD47 [139]. *Bifidobacterium bifidum* can migrate to and colonize the tumor site, activate DCs, and enhance the local immune response. Systemic administration of *Bifidobacterium* caused non-responsive mice to respond to anti-CD47 treatment, and local delivery strongly stimulated STING signaling, further enhancing DC activation. This reveals how gut microbes promote tumor immunotherapy via the STING signaling pathway. Lam et al. showed that microbial-derived STING agonists (c-di-AMP) enhance anti-tumor immune responses by inducing tumor-tissue monocyte-derived interferon type I (IFN-I) production, and modulating macrophage polarization and NK-DC interactions. In addition, *A.muciniphila*-derived STING agonists have improved the efficacy of ICIs in melanoma patients [140].

## Tumor microbiome and metastasis

### Mechanisms of tumor metastasis and colonization

Mechanistically, the hypothesis regarding tumor metastasis has evolved over the past few decades. A series of processes occur sequentially within the TME to promote metastasis, each playing a critical role in the successful spread of tumor cells. To begin with, the initial establishment of specific cell adhesion initiates the whole process, in which tumor cells form stable attachments to surrounding stroma or cells. Next is autonomous migration, where tumor cells infiltrate adjacent tissues and degrade the extracellular matrix, allowing them to move through the local environment. The third step is entry into the bloodstream or lymphatic system (intravasation), where tumor cells must survive unfavorable conditions and exit the blood stream (extravasation). Once surviving tumor cells extravasate and migrate to distant tissues, they tend to form pre-metastatic niches (PMNs), preparing tumor cells for metastatic colonization and establishing secondary tumors in the new organ [141, 142]. Therefore, a successful metastatic route usually involves a complex series of interactions among various cell types. Notably, EMT, one of the most recognized features of malignancy, can also promote the metastatic process. The relationship between EMT and tumor cell metastasis lies in its ability to endow tumor cells with stronger mobility and invasiveness, enabling them to detach from the primary tumor and enter the bloodstream or lymphatic system. During EMT, tumor cells lose polarity, acquire apoptosis-resistant traits, and gain enhanced migratory and invasive capabilities [143].

The prerequisite of tumor cells relocation is detachment from the primary sites, to realize this step, tumor cells need to reduce intercellular connections through EMT, which allows them to detach from the primary lesions and initiate the directional motility. Directed migration usually depends on cytoskeletal remodeling, dynamic interactions with the ECM, and specific cellular adhesion molecules, such as cluster of differentiation 44 family (CD44 family), cadherins, integrins, selectins, and immunoglobulin superfamily [144–147]. More specifically, this involves eliminating obstacles created by ECM components through chemical degradation, which can be regarded as the dominant motility strategy for cell migration in the metastatic cascade process [148]. Chemical degradation refers to mesenchymal migration that depends on protease-induced ECM degradation, primarily involving MMPs. This process is characterized by tumor cells completing adhesion through actin cytoskeleton remodeling, followed by invasive environmental remodeling and contraction, independent of cell polarization [149]. Once ECM is degraded, tumor cells can achieve the directed migration in the form of amoeboid movement. Driven by chemotactic cytokines, tumor cells can move by extending and contracting pseudopodia, creating a path where ECM attachment is loose and migratory constraints are minimal [150]. Moreover, when tumor cells migrate into the circulatory system, they undergo a series of morphological adjustments to enhance their migratory capabilities, including the formation of specialized adhesive structures called invadopodia, primarily expressed in the membranes of metastatic cells. Invadopodia contain a high concentration of proteolytic enzymes, with MMP14, a transmembrane protein in the MMPs family, being predominantly expressed [151]. Additionally, actin-dependent pseudopodia protrusions are key driving factors in the EMT process, highlighting the importance of EMT in tumor cell metastasis [152].

Once directional motility is established, tumor cells are likely to attach to endothelial cells. This process involves receptor-ligand interactions, activation of signaling pathways, and the participation of platelets and white blood cells, which determine whether tumor cells can successfully attach, cross the endothelial barrier, and ultimately form circulating tumor cells (CTCs) [148]. Some tumor cells develop a tendency to secrete pro-apoptotic signals to induce endothelial cell apoptosis during transmigration [153]. Concurrently, tumor cells can weaken the endothelial barrier by secreting factors that promote endothelial cell contraction and disrupt cell connections, such as VEGF and transforming growth factor beta (TGF-β) [154]. After intravasation into the vasculature, tumor cells that enter the bloodstream become CTCs. To successfully colonize distant sites, CTCs must endure fluid shear stress, which likely reduces their survival rates and decreases the probability of metastasis. This stress may also trigger receptor-mediated apoptosis in tumor cells independently of apoptotic agents [155, 156]. Additionally, elevated ROS present another challenge for CTCs. As a byproduct of cellular respiration, ROS is primarily produced in mitochondria, recognized as the main site of ROS generation [157]. Continuous ROS accumulation can enhance tumor cells’ capacity for mutation, invasion, and metastasis [158]. However, Piskounova et al. found that the metastatic site selection partly depends on the ability of CTCs to withstand oxidative stress, as observed in melanoma mouse models, suggesting that ROS can restrict metastasis through multiple known pathways [159]. Additional explanations for this phenomenon include that elevated ROS levels may induce ferroptosis, characterized by lipid peroxidation [160].

Metastasis is also regulated by systemic processes like immune surveillance, where innate and adaptive immune cells, including NK cells, macrophages, and lymphocytes, play crucial roles in eliminating CTCs. As part of the innate immune system, NK cells can directly kill CTCs by releasing cytotoxic molecules like perforin and granzyme [161]. NK cells can also compete with platelet aggregates on the CTC surface, counteracting the defenses that tumor cells employ [161]. Additionally, NK cells can secrete IFN-γ and TNF-α to activate T and B cells, enhancing adaptive immunity [162]. EMT is associated with the upregulation of various natural killer group 2 member D (NKG2D) ligands and the downregulation of NK cell inhibitory receptor ligands, such as E-cadherin, which makes potential metastatic cells more easily recognized and eliminated by NK cells [163, 164]. However, using single-cell RNA sequencing and spatial transcriptomics, Mao C et al. revealed that resting NK cells can promote liver metastasis in colorectal cancer [165]. This finding suggests a complex relationship between NK cells and their anti-tumor effects. For instance, one reason tumor cells can still metastasize despite the presence of a fully functional NK system is their ability to induce platelet aggregation, which acts as a protective mechanism [166]. Specifically, as the first point of contact with CTCs, platelets protect tumor cells through aggregation-induced surface shielding, guarding CTCs from NK cell-mediated cytotoxicity. Moreover, tumor cells can activate platelets to mediate endothelial barrier disruption via P2Y2 receptors, promoting tumor cell metastasis [167]. Compared with NK cells, CD8 + T cells rely on correctly binding to major histocompatibility complex class I (MHC I) expressed by abnormal mutant cells to effectively eradicate them by producing cytotoxic granules [168]. Given their delayed responsiveness and lower attack sensitivity, adaptive immunity contributes less to the elimination of CTCs, which have evolved advanced mechanisms for evading immune surveillance, such as epitope escape, immune inhibition, and apoptosis induction [169].

**Fig. 6:**
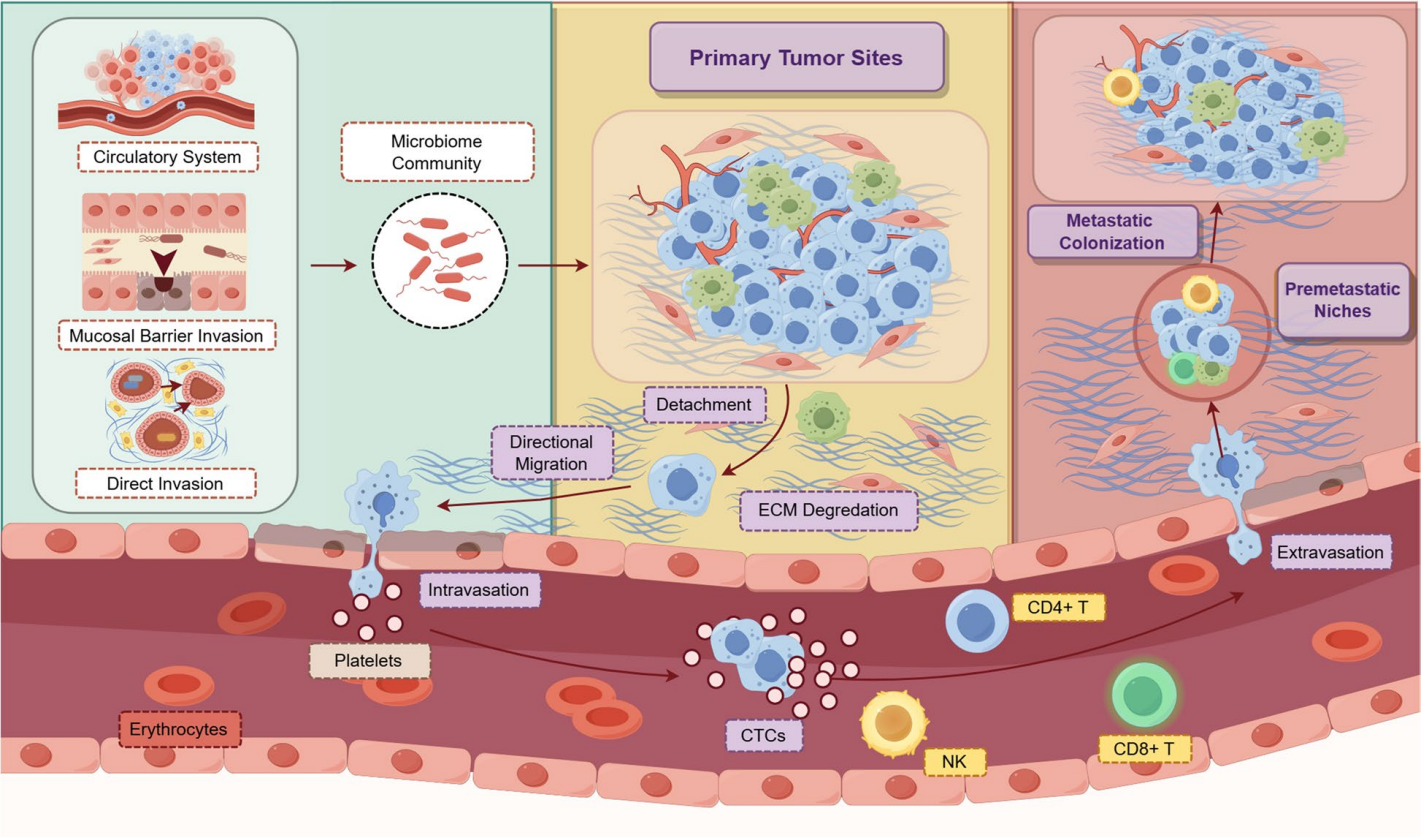
The process of tumor cells migrating from the primary tumor site to distant metastatic sites with microbiota involvement. The left side of the figure outlines the origins of tumor-associated microbiota, entering the circulatory system through hematogenous spread or lymphatic drainage, and invading tissues via disrupted mucosal barriers or direct penetration. The blue boxed text represents steps in tumor metastasis: tumor cells detach from primary sites, infiltrate adjacent tissues, and degrade the extracellular matrix; they then enter the bloodstream as CTCs. Shielded by platelets, CTCs survive immune attacks and evade immune detection to extravasate at distant endothelial sites, forming premetastatic niches and eventually colonizing secondary sites. (Generated using Figdraw)

Furthermore, selected CTC clusters initiate adhesion to endothelial walls at specific sites, similar to leukocyte extravasation in inflamed tissues [153]. This process provides a model for the extravasation route of tumor migration: the ‘outpost’ cells adhere dynamically to the vascular endothelium, then proceed through the vascular walls to reach the surrounding matrix and colonize secondary lesions. During this process, members of cell adhesion molecules (CAMs) family—such as integrins, cadherins, and the immunoglobulin superfamily—engage through interactions with the ECM [170]. To extravasate from circulation, tumor cells follow an inevitable path through transmembrane passage, where their comparatively larger size often causes them to become trapped in microvessels. Mechanistically, these trapped tumor cells undergo membrane deformation or rupture, releasing substances that damage surrounding capillaries, creating space for other CTCs to exit blood vessels [171]. Alternatively, extravasation can be mediated by selectin families, which play a pivotal role in regulating the process by dynamically controlling the rolling and adhesion of tumor cells to the vasculature [172]. Notably, there is clear evidence linking cytoskeletal rearrangements to endothelial retraction, which serves as an entry point for tumor cell extravasation through transendothelial migration, mediated by Ras homologous guanosine triphosphatases (Rho GTPases))) [173]. Consequently, once CTCs that have not been fully eradicated transmigrate from the vasculature to the ECM, they infiltrate and colonize preferred organs, forming metastatic lesions. The general mechanism concerning on the metastasis has been elucidated in Fig. 6.

### Microbial influence on the metastatic cascade

Successfully colonizing distant organs requires tumor cells to undergo a series of intrinsic and extrinsic changes. This occurs when metastatic cells interact with dynamic TME components, allowing them to survive and settle in specific niches. With the emergence of the tumor microbiome, researchers have gradually revealed its role in potentiating metastatic effects [46]. Reciprocally, tumor-related microorganisms play detrimental roles in metastatic characteristics through complex mechanisms, primarily by modifying the intrinsic microenvironment and reshaping the extrinsic TME. Theoretically, the tumor microbiome might influence any of the critical steps in metastasis, potentially triggering cascades that impact the entire metastatic process, especially during key stages such as invasion, circulation, and colonization at distant sites [174]. The impact of the tumor microbiome on metastasis process across various malignancies is summarized in Table 2. Given the distinct characteristics of each type, the most commonly metastasizing cancers include colorectal, breast, lung, and head and neck cancers. Based on these findings, we will focus on detailed mechanisms by which microbes influence the stages of metastasis.

**Table 2:** Studies on microbiota-induced tumor metastasis and the underlying mechanisms

Malignancy Type	Microbiome Strain	Mechanism Contributing to Tumor Metastasis	Detection Technology	Ref
Colorectal Cancer	*F. nucleatum, P. intermedia*	Collaboratively promote adenoma-carcinoma transformation and cancer metastasis	16S rRNA Sequencing	[175]
Colorectal Cancer	*F. nucleatum, Bacteroides, Selenomonas,* and *Prevotella*	Constant presence both in the primary and metastatic sites	16S rRNA Sequencing, qPCR, RNA-seq, FISH, Culture	[21]
Colorectal Cancer	*F. nucleatum*	Promote EMT and cancer metastasis by activating the TLR4/Keap1/NRF2 axis, based on both CYP2J2 and 12,13-EpOME-induced metabolic modifications	qPCR, FISH, IHC, Metagenomic sequencing	[176]
Colorectal Cancer	*F. nucleatum*	Promote tumor metastasis dependent on miR-1322/CCL20 axis and M2 macrophage polarization	16S rRNA Sequencing, RT-qPCR, RNA-Seq	[177]
Colorectal Cancer	*F. nucleatum*	Enhance MMP7 expression mediated by MAPK(JNK)-AP1 axis, thus promoting metastasized steps	16S rRNA Sequencing, qPCR	[178]
Colorectal Cancer	*F. nucleatum*	Facilitate tumor metastasis by inducing ICAM1 expression	RNA-seq, qPCR, IHC, Culture	[179]
Colorectal Cancer	*F. nucleatum*	Mediate exosomes releases that comprise certain carrying miR-1246/92b-3p/27a-3p and CXCL16 to enhance metastasis	RT-qPCR, Proteomics, IHC, Culture	[180]
Colorectal Cancer	*E. coli*	Induce premetastatic niches formation through disrupting gut vascular barrier	16S rRNA Sequencing, FISH, RT-qPCR, Culture, whole-genome sequencing	[181]
Esophageal Cancer	*F. nucleatum*	Increase METTL3-mediated m6A methylation to enhance metastasis	qRT-PCR, IF	[101]
Breast Cancer	*S. xylosus, L. animalis, S. cuniculi*	Suppress CTCs elimination through actin cytoskeleton rearrangements to promote metastasis	16S rRNA Sequencing, qPCR, FISH, Culture, IHC, TEM	[22]
Breast Cancer	ETBF	Secrete toxin *Bft* to facilitate migratory and invasive ability of breast cancer cells, thus enhancing the metastatic potential, on the basis of both β-catenin and Notch1 pathway uplifting	16S rRNA Sequencing, qPCR, RNA-Seq, IHC	[182]
Breast Cancer	*F. nucleatum*	Utilize Fap2 to bind to increased Gal-GalNAc in tumor cells preferentially, thus diminishing the infiltration of CD4 + /CD8 + T cells and promoting tumor progression and metastasis	16S rRNA Sequencing, qPCR, IHC, Flow Cytometry	[183]
Liver Cancer	*Clostridium* genus	Regulate chemokine expression and NKT cell accumulation through modulating bile acid metabolism, thereby affecting the antitumor immunity and tumor metastasis process	16S rRNA Sequencing, IHC, RT-qPCR	[123]
Pancreatic Cancer	*Comamonas* and *Turicibacter*	Highly rich in tumor samples with lymph nodes metastasis	16S rRNA Sequencing, RT-qPCR	[184]
Lung Cancer	*Roseburia*	Promote lymph node metastasis through secreting butyrate, which can inhibit HDAC2, increase H3K27 acetylation at the H19 promoter, as well as induce M2 macrophage polarization	16S rRNA Sequencing, RT-qPCR, IHC	[102]
Lung Cancer	*Proteus* and *Bacteroides*	Upregulate cell cycle and energy metabolism pathways, which can promote tumor metastasis	RNA-seq, GSEA	[185]
Lung Cancer	*Legionella*	Exhibit a parallel elevation in line with tumor metastasis progression	16S rRNA Sequencing	[186]
Lung Cancer	*P. gingivalis*	Promote lung cancer metastasis and progression through long-term colonization	IHC	[39]
Oral Cancer	*F. nucleatum*	Produce OMVs to enhance metastasis through EMT and corresponding autophagy	TEM, RNA-seq, RT-qPCR, FISH	[187]
Oral Cancer	*Prevotella, Stomatobaculum, Bifidobacterium,* and *F. nucleatum*	Serve as the biomarker to predict the lymph node metastasis	16S rRNA Sequencing	[188]
Oral Cancer	*P. gingivalis*	Enhance the invasive and metastatic ability by inducing EMT and promoting MMP1/10 expression	16S rRNA Sequencing, RT-qPCR, IF, Flow Cytometry	[189]
Oral Cancer	*P. gingivalis, F. nucleatum*	Promote tumor metastasis through elevated MMP9 gene expression, triggered by TLR signaling and subsequent IL-6 production and STAT3 activation	16S rRNA Sequencing, RT-qPCR, IHC	[57]
Laryngeal Squamous Cell Carcinoma	*F. nucleatum*	Promote tumor metastasis by reprogramming ethanol metabolism and activating the PI3K-AKT signaling pathway associated with patients poor prognosis	16S rRNA Sequencing, FISH, IHC	[80]
Nasopharyngeal Carcinoma	*Corynebacterium* and *Staphylococcus*	Suppress T-lymphocyte infiltration to promote metastasis	16S rRNA Sequencing, FISH, Metagenomic sequencing, RT-qPCR, Culture	[190]

*Abbreviations*: *P. intermedia Prevotella intermedia*, *S. xylosus Staphylococcus xylosus*, *L. animalis Lactobacillus animalis*, *S. cuniculi Streptococcus cuniculi*, *Kep1* Kelch-like ECH-associated protein 1, *Nrf2* nuclear factor erythroid 2-related factor 2, *CYP2J2* cytochrome P450 epoxygenase 2J2, *12,13-EpOME* 12,13-epoxyoctadecenoic acid, *miR* microRNA, *CCL20* chemokine (C–C motif) ligand 20, *MMP7* matrix metalloproteinase 7, *JNK* c-Jun N-terminal kinase, *AP1* activator protein 1, *ICAM1* intercellular adhesion molecule 1, *Notch1* notch homolog 1, *OMVs* outer membrane vesicles, *GSEA* gene set enrichment analysis

#### Tumor invasion

As mentioned above, tumor cells must invade surrounding tissues after detaching from primary sites. During this process, they enhance their invasive capacity through intrinsic and extrinsic modifications. MMPs play an essential role in the degradation and remodeling of the ECM, eliminating obstacles through chemical breakdown [191]. They are of great significance for tumor cell dissemination, along with promoting EMT, modulating the immunity, and influencing the angiogenesis [192]. Driven by metastasis-promoting factors, MMP7 has been identified as expressed in *F. nucleatum*-infected CRC cells, promoting metastatic progression. Here, MMP7 was presumed to enforce tumor extravasation through clearing E-cadherin, eliminating relevant obstacles, and endowing them with more accessibility traits of detaching from the primary lesions. Mechanistically, the MAPK(JNK)-AP1 axis regulates MMP7 expression at the transcriptional level [178]. Besides, *F. nucleatum* can also promote CRC invasion through immunity and cellular adhesion, which included upregulating the inflammatory chemokines such as CCL20 and C-X-C motif chemokine ligand (CXCL8) and interfering with cell adhesion functions [178]. *P. gingivalis* was detected to induce the elevated levels of both MMP1 and MMP10, which are triggered by the release of interleukin-8 (IL-8) [189]. Moreover, triggered by TLR signaling and subsequent IL-6 production and STAT3 activation, both *P. gingivalis* and *F. nucleatum* contributed to oral cancer progression and metastasis through the elevated MMP9 gene expression, running parallel to cyclinD1 and heparinase [57]. Likewise, a decrease in E-cadherin and an increase in neural cadherin (N-cadherin) can also be seen in oral cancer, infected by *F. nucleatum* [187].

Additionally, Zhang Y et al. proposed that *F. nucleatum* promoted intercellular adhesion between tumor cells and endothelial cells, thereby enhancing the invasion and extravasation ability of malignant cells [193]. In concordance to what has been elucidated above, CAMs present an indispensable intersection in regulating the tumor cell detachment, extravasation, migration, and metastasis, and a significantly enhanced levels of mRNA and proteins of ICAM1, which contributed to intercellular communication as the trans-membrane protein mostly expressed in vascular endothelial cells. It demonstrated that CRC cells infected by *F. nucleatum* are more prone to adhering to the vascular walls on the metastatic environment. Moreover, enhancing the migration ability of CRC was also revealed to be a significant trait of *F. nucleatum,* helping CRC cells pass through the vascular wall and form metastatic lesions in distant organs [194]. This reinforced propensity was linked to the activation of the NF-κB signaling pathway and depended on higher levels of alpha-kinase 1 (ALPK1) expression, accountable for recognizing molecular structures of *F. nucleatum* surfaces as one of pattern recognition receptors [193]. Therefore, the ALPK1/NF-κB/ICAM1 axis mediated this whole process [179].

#### Circulation

A prominent route for metastatic tumor colonization is circulation, where tumor cells encounter shear stress and often bind to platelets to form a protective barrier [167]. However, certain microbial communities reinforce CTC cells resistance to external fluid forces, enabling them to withstand mechanical stresses during transit, which would otherwise lead to apoptosis [156]. Hence, resistance to external fluid forces in the vessels, combined with the internal modifications to alleviate stress, is crucial for achieving successful seeding in distant sites [195–197]. In this context, seminal studies have examined the role of the tumor-intrinsic microbiome in breast cancer. Using in vitro fluid shear stress simulations, Fu A et al. revealed that CTCs invaded by *S. xylosus, L. animalis,* and *S. cuniculi* promoted metastasis through actin cytoskeleton rearrangements, which reduced the intensity of mechanical stress [22]. For specific targeting, cell-penetrating antibiotics were administered to confirm that tumor-resident bacterial communities could travel with host cancer cells to distant organs for specific migration purposes [22]. With retrospect to the original description, the cytoskeletal remodeling featured with EMT was also explored to be the means of shielding from extracellular stimuli and aiding in metastasis [196, 198].

Detrimentally, another factor promoting metastasis in breast cancer is toxin-related migration. Certain bacteria produce specific toxins that facilitate tumor cell migration. *B. fragilis*, commonly found in the breast, colon, and oral cavity as part of the microbiome, typically helps maintain immune homeostasis. However, as revealed by Sheetal P et al., ETBF, as a toxin-producing substrain, was proven to significantly enhance metastasis in breast cancer cells [199, 200]. ETBF-infected tumor cells acquire highly migratory and invasive abilities, along with increased expression of β-catenin and activation of the Notch1 pathway through toxin-induced mechanisms. This increased metastatic tendency underscores ETBF’s role in promoting breast cancer cell migration and secondary colonization [199]. Similarly, studies have noted that the chemokine IL-17 as the inhibitor of CD8 + T cell and Treg (Regulatory T cell) infiltration, thus promoting colorectal malignancies [201]. However, whether this chemokine facilitates metastasis and migration in colorectal cancers remains uncertain and warrants further investigation.

#### Tumor-related immunity

The incidence of malignancy likely induces immune stimulation. As a key component of the TME, immune cells play a pivotal role in promoting metastasis [202]. Among the various immune cells associated with malignancy, macrophages are most closely linked to metastasis [203]. Within the TME, TAMs continuously transition between M1 and M2 polarization states [204]. M1 macrophages foster cytotoxicity and create a tumor-suppressive microenvironment, while M2 macrophages promote immunosuppression and secrete cytokines that support tumor survival and growth. In colorectal cancer, for instance, *F. nucleatum* enhances macrophage infiltration and promotes the polarization of macrophages towards the M2 phenotype in a CCL20-dependent manner, contributing to tumor metastasis [177]. CCL20, a specific chemokine regulated by miRNA-1322, is crucial for tumor metastasis and progression. Simultaneously, kyoto encyclopedia of genes and genomes (KEGG) and gene set enrichment analysis (GSEA) analysis indicated that miRNA-1322 activation was mediated through the NF-κB pathway, suggesting relevant transcription profiles [177]. This finding implies the fact that chemokines intersect across various pathways between tumor cells and normal tissues [205]. Given their chemotactic roles, chemokines are believed to attract specific immune cells and mobilize their differentiation [206]. Furthermore, cytokines are sometimes secreted in the form of extracellular vesicles (EVs). To effectively promote metastasis, tumor cells must actively communicate with target cells in the TME. During this process, they are thought to secrete EVs carrying bioactive factors (DNA, RNA, proteins, lipids, and metabolites), whose membrane components originate from the parent cells [207]. Once transported to recipient cells, EVs initiate a series of programmed cellular activities in the TME, highlighting their crucial role in mediating communication across heterogeneous tumor cells [208, 209]. Similarly, infection by *F. nucleatum* leads to an increased abundance of tumor-derived EVs, which carry specific miRNAs (miR-1246, 92b-3p, 27a-3p) and protein cargos (CXCL16, RhoA, and IL-8) to non-infected cells, enhancing their migratory capabilities [180]. In CRC, research has shown that high expression of the chemokine receptor CCR6 in T cells is closely related to liver metastasis [210]. An increase of CXCL16 is secreted is likely to facilitate the homing of non-infected recipient CRC cells to the hepatic vessels by the CXCL16/CXCR6 axis [180]. Research has also shown that *Clostridium* species enhance the hepatic antitumor immune response by regulating bile acid metabolism. To be more specific, *Clostridium* modifies bile acids to signal liver sinusoidal endothelial cells to produce CXCL16, which promotes the accumulation of CXCR6 + NKT cells [123].

In terms of the adaptive immunity, Parhi L et al. proposed that *F. nucleatum* colonized the breast tumors cells by binding its protein Fap2 to the Gal-GalNAc on the surface of tumor cells. This colonization inhibited the infiltration of immune cells, especially T cells, leading to enhanced tumor growth and metastasis in an immune-context measure [183]. The abundance of *F. nucleatum* is predominantly observed in samples with high Gal-GalNAc levels, a biomarker associated with precancerous lesions [211]. It was believed that *F. nucleatum* communities were trafficking in a Fap2-binding dependent manner when supplied with abundant blood circulation from the primary resident niches [183]. Additionally, investigators found that high bacterial loads in nasopharyngeal carcinoma are negatively associated with CD8 + T cell infiltration, which is crucial for suppressing tumor invasiveness by interacting with the TME as a key immune surveillance agent [212]. The subsequent diminishing amount of CD8 + T cells responsible for eliminating tumor cells was possibly one of the contributing factors causing the metastasis [190]. These above effects suggests that bacterial colonization within tumors may promote the formation of a favorable microenvironment for tumor progression by altering immune surveillance and supporting tumor cell survival and spread.

#### PMNs

PMNs are defined as a specific spatial environment shaped in distant organs from primary tumors. PMNs provide a favorable environment for tumor metastasis, serving as a prerequisite for CTC colonization at secondary lesions. To understand the formation of PMNs, it is crucial to focus on specific premetastatic sites. Research has shown that vascular endothelial growth factor receptor 1-positive (VEGFR1 +) hematopoietic precursor cells derived from bone marrow form cell clusters at premetastatic sites, providing a microenvironment for tumor metastasis [213]. Additionally, recent studies have shown that, aside from bone marrow-derived precursor cells, certain microbiome communities can also subtly impact tumor metastasis by inducing PMN formation. For instance, *E. coli* can potentially facilitate PMN formation by disrupting the gut vascular barrier (GVB) [181]. To be more specific, *E. coli* facilitates the transfer of bacterial communities from the gut to the liver by disrupting GVB. This disruption, mediated by a virulence factor (VirF) in *E. coli*, creates a PMN in the liver by recruiting immune cells and fostering an inflammatory environment [181]. This phenomenon highlights a mechanism where gut microbiota contributes to tumor spread by altering vascular permeability. Changes in the gut microbiota can increase the permeability of the intestinal barrier, prompting bacteria to enter the liver and form a pre-metastatic microenvironment [214]. Therefore, as mentioned above, the gut microbiome possesses the capability of promoting the tumor seeding in pre-metastatic organs [215]. Future research should further explore how specific microbial communities affect PMN formation in different organs through various mechanisms, providing potential pathways for targeted microbiota-based anti-metastatic therapy.

### EMT

From an intrinsic perspective, EMT is characterized by an augmented metastatic propensity of tumor cells, accompanied by enhanced migratory and invasive abilities, reduced adhesion, cytoskeletal reorganization, and resistance to apoptosis [143]. Therefore, since EMT promotes tumor cell metastasis in nearly every aspect, some microbiomes that can induce EMT in tumor cells are often associated with metastatic potential. For instance, in laryngeal squamous cell cancer, Hsueh CY et al. proposed that *F. nucleatum* enhanced the migration and invasion ability of tumor cells by regulating the expression of EMT-related genes, such as reducing the expression of E-cadherin and increasing the expression of N-cadherin and Vimentin [80]. *P. gingivalis* can induce morphological changes in oral squamous cell carcinoma cells, thereby enhancing cancer cell migration, invasion ability, and tolerance to chemotherapy drugs [189]. In oral cancer, it has also been revealed that OMVs of *F. nucleatum* can activate autophagy in cancer cells and induce EMT to promote cancer metastasis [187].

Furthermore, EMT is subtly correlated with the metabolic modifications. In colorectal cancer, research found that *F. nucleatum* promotes metastasis by upregulating CYP2J2 and its metabolite 12,13-EpOME, which enhances cancer cell invasiveness via the TLR4/Keap1/NRF2 axis and EMT [176]. Studies have found that the elevated abundance of *Tepidimonas* and its secreted metabolites in pancreatic cancer can promote EMT, enhancing the proliferation and migration of pancreatic cancer cells. *Tepidimonas* activates EMT-related signaling pathways and alters tricarboxylic acid cycle metabolites, driving cancer cell metabolic changes and increasing tumor invasiveness, indicating its role in promoting pancreatic cancer metastasis [184]. EMT-related genes, such as E-cadherin and vimentin, are highly expressed when *E. coli* is enriched in bladder cancer [216]. Therefore, EMT is an important bridge between tumor microbiota and cancer metastasis, this process involves multiple signaling pathways. Undoubtedly, EMT intersects across a wide range of metastasized steps, which may become effective therapeutic targets for advanced cancer management.

## Microbiome-driven resistance to tumor therapies

### Microbiome-based tumor therapeutics modulation

The effectiveness of tumor treatment largely depends on how patients respond, including their sensitivity to chemotherapy, radiotherapy, and immunotherapy. This section examines the microbiome’s impact on these key therapeutic approaches, offering a deeper understanding of its role in drug resistance and its potential in shaping future treatment strategies.

#### Chemotherapy

Notably, chemotherapy drug resistance in tumor patients is one of the leading factors contributing to tumor recurrence and progression [217]. To the best of our knowledge, possible reasons causing tumor drug resistance include drug inactivation, enhanced drug efflux pump activity, gene instability, epigenetic changes, inhibition of apoptosis protein overexpression, and EMT, which collectively reduce the effectiveness of treatment and promote metastasis [218].

Given the dynamic interaction between the microbiome and the TME, certain microbial communities can subtly modulate chemotherapy efficacy by enhancing drug effects, suppressing anti-tumor activity, or mediating toxicity [219]. For instance, Daillère R et al. proposed *Enterococcus hirae* and *Barnesiella intestinihominis* could enhance the anti-tumor efficacy of Cyclophosphamide (CTX) through different mechanisms respectively. The former translocates from the small intestine to secondary lymphoid organs, increasing CD8 + T cell proportions in the TME and restoring chemotherapeutic efficacy. The latter, accumulating in the colon, enhances the antineoplastic effects of CTX by promoting γδT cell infiltration and INF-γ production, collectively improving chemotherapy responsiveness [220]. In a similar manner, CTX has been found to stimulate specific immune responses by altering the composition of the small intestinal microbiota and promoting the transfer of gram positive bacteria to lymphoid organs. Aseptic or antibiotic-treated mice developed CTX resistance, while transplantation of pathogenic T-helper 17 cells partially restored their antitumor effects [221]. Another study revealed that healthy gut microbiota can enhance the efficacy of anti-tumor drugs by promoting the release of ROS from myeloid cells and strengthening inflammatory responses, and this effect will decrease under antiobiotics administration in germ-free mice [222].

#### Radiotherapy

Radiotherapy is considered the standard treatment for most malignancies, but it often exhibits a range of severe side effects. The efficacy of radiotherapy often depends on the patient’s tolerance level [223]. Given that the microbiome community plays a crucial role in regulating systemic immune responses, which can affect radiation responses and gastrointestinal toxicity, targeting side effects is crucial to achieving an effective radiotherapy [224]. In recent years, research has explored the potential of using microbiomes as mediators to address these challenges.

For instance, *Lachnospiraceae* and *Enterococcus* microbiota improved radiotherapy efficacy by promoting hematopoietic function and gastrointestinal repair [225]. The metabolites of these microbial communities can alleviate radiation-induced damage and reduce side effects, such as fatigue, nausea, vomiting, and diarrhea [225]. For instance, both *Lactobacilli* and *Bifidobacteria* have been shown to reduce diarrhea-related side effects, and enhance radiotherapy tolerance by regulating immune responses [226]. Besides, bacterial-based immunotherapy can exert a synergistic effect in eliminating tumors by enhancing the efficacy of radiotherapy. CpG Oligonucleotides (ODN) is a synthetic DNA molecule that can regulate immune responses by activating TLR9 [227]. Research has shown that CpG ODN has a sensitizing effect in radiotherapy, it can improve the efficacy of radiotherapy by regulating the immune system, with concurrently reducing radiation damage, alleviating oxidative stress and inflammatory responses caused by radiotherapy [228]. Furthermore, microbiome communities shifts in the gut can affect the radiotherapy resistance, the reduction in bacterial species in microbial composition can cause a decrease in host tolerance to radiotherapy [229]. Besides, total-body radiation (TBI) enhances tumor immune response through mediating microbiome migration towards the mesenteric lymph nodes, accompanied by an increase in serum levels of lipopolysaccharides, thus promoting tumor regression [120].

#### Immunotherapy

Immunotherapy is increasingly recognized as a standardized treatment modality due to its promising efficacy [230]. However, with an overwhelming number of terminal patients who fail to respond durably to immunotherapy, it is urgent to look into the microbial colonization with practical utility in tumor management, especially ICIs such as PD-1/PD-L1 inhibitors and cytotoxic T-lymphocyte associated protein 4 (CTLA-4 blockades), to reactivate anti-tumor immunity.

Studies have shown that the anti-tumor effect of CTLA-4 blockade therapy depends on specific *Bacteroides* species, especially *Bacteroides thetaiotaomicron* and *B. fragilis*, which can enhance tumor immune responses by promoting T cell activation and tumor-infiltrating lymphocyte function [231]. Additionally, other microorganisms, especially *Bifidobacterium*, can enhance the efficacy of PD-L1 therapy. Experiments have shown that mice with different gut microbiomes exhibit differences in melanoma growth and spontaneous anti-tumor immune responses. Gavage of *Bifidobacterium* in mice significantly improved tumor control, and combined with PD-L1 antibody, almost completely inhibited tumor growth [232]. *Bifidobacterium* can improve the efficacy of ICIs significantly through enhancing the function of DCs, activating CD8 + T cells, and stimulating IFN-γ production [232]. The presence of *A. muciniphila* in renal carcinoma has also been witnessed to improve clinical effectiveness of patients undergoing anti-PD-1 treatment [60]. In pancreatic ductal adenocarcinoma, 16S rRNA sequencing of patient samples revealed an enriched bacterial community, *Megasphaera*, which may enhance anti-tumor immunity by producing butyrate and synergizing with ICIs to improve therapeutic efficacy. However, compared to previous findings, butyrate, as one of the most studied short-chain fatty acids, exhibits dual roles in tumor-related immunity, highlighting the complexity across different tumor types [233]. Moreover, Chu S et al. revealed that airway-enriched *F. nucleatum* before anti-PD-1 therapy contribute to a poor response in lung cancer [234]. Therefore, further research is needed to fully understand the underlying mechanisms of microbiome-based interventions to optimize their use in clinical practice.

### Recent insights on microbiome-mediated drug resistance

#### Immunity

The role of host immunity in chemotherapy resistance is multifaceted, particularly through regulating the TME and immune cell function. To be more specific, research has found that *F. nucleatum* promotes oxaliplatin resistance in CRC patients by activating the innate immune system. Specifically, *F. nucleatum* activates the autophagy pathway by regulating the TLR4 and MYD88 signaling pathways and specific microRNAs, thereby promoting chemotherapy resistance [235]. Besides, similar articles suggest that *F. nucleatum* can also inhibit the CD8 + T cells and NK cells by increasing pro-tumor immune cells such as neutrophils, DCs, and M2 macrophages, thereby weakening the host's immune response, with concurrently activating autophagy pathways [236]. Under anaerobic conditions, *Raoultella planticola* can inactivate doxorubicin through deglycosylation, reducing its toxicity, while *K. pneumoniae* and *E. coli* can also degrade the drug through molybdenum cofactor enzymes [237].

#### Drug metabolism

Surprisingly, bacterial communities can metabolize chemotherapy drugs, thereby affecting their activity and local concentration. For instance, by using 16S rRNA sequencing on samples extracted from tumor patients, researchers found *Gammaproteobacteria*, enriched in PDAC and most of them are members of the *Enterobacteriaceae* and *Pseudomonadaceae*, can induce gemcitabine resistance by metabolizing gemcitabine into an inactive form through expressing a long isoform of cytidine deaminase (CDDL) [238]. Additionally, *Lactobacillus iners* alters the metabolic pathway of tumor cells by enhancing the lactate signaling pathway, regulating oxidative stress and hypoxia inducible factor 1 related pathways, thus leading to resistance to chemotherapy and radiotherapy [239].

#### Anti-apoptotic pathways

The overproliferation of anti-apoptotic pathways is one of the universal traits of malignancy. BIRC3 as a gene encoding an inhibitor of apoptosis protein, can prevent cell apoptosis by directly inhibiting pro apoptotic caspases or by regulating cell survival and inflammatory pathways [240]. Overexpression of BIRC3 is closely related to chemoresistance. Research has found that *F. nucleatum* infection upregulates BIRC3 gene expression through the TLR4/NF-κB pathway, reducing CRC cell sensitivity to 5-Fluorouracil and contributing to chemotherapy resistance [241].

## Strategies for targeting microbiome in tumor therapy

### Engineered/Non-engineered bacteria-based therapies

The impact of microbial communities on tumor invasion, progression, and metastasis reveals the potential of microbiome-derived enzymes and proteins in tumor treatment. Microbiome-based therapy has gradually become an emerging treatment method, mainly by directly activating anti-tumor immune responses through various pathways or indirectly acting as a tumor vaccine [242]. Due to the irreplaceable advantages and characteristics of bacterial communities, which can assist in tumor treatment by regulating immune responses (as tumor vaccines), influencing metabolic microenvironment, delivering therapeutic drugs, and inducing cell apoptosis, innovative approaches have opened new avenues for tumor therapy [242].

Bacillus Calmette-Guérin (BCG), a live attenuated strain of *Mycobacterium bovis* with a history of over a century, still remains a prime example of microbial therapy not only used in tuberculosis prevention, but also treating non-muscle invasive bladder cancer (NMIBC) [243]. This case holds milestone significance in the use of exogenous bacteria for disease treatment. Moreover, systemic administration of microbiomes which can deliver therapeutic molecules can reach a potent efficacy in tumor treatment. A study introduced that *Salmonella typhimurium* (VNP20009), an attenuated strain coated with antigen-absorbing nanoparticles, when injected into tumors after radiotherapy, can activate T cells by delivering tumor antigens to DCs, thereby triggering a systemic anti-tumor immune response. This approach, especially when combined with ICIs, effectively enhances anti-tumor immunity, offering a new direction for tumor vaccine development [244].

From the metabolic landscape in TME, *E. coli*-derived asparaginase has been used for remission induction and intensification treatment in acute lymphoblastic leukemia for a prolonged period, mainly through depleting asparagine [245]. Likewise, *Mycoplasma* serves as a source of anti-tumor enzymes, and its arginine deiminase (ADI) is used for amino acid deprivation therapy to inhibit tumor growth [246]. Moreover, *Mycoplasma* can also induce apoptosis of tumor cells, especially in arginine dependent tumors such as melanoma and hepatocellular carcinoma, by consuming arginine that tumor cells rely on for survival. ADI derived from *Mycoplasma* can eliminate melanoma cells by activating apoptotic pathways dependent on caspase-8 and caspase-10. This mechanism makes it a promising anti-tumor strategy [246]. Furthermore, given that bacterial communities have a natural tendency to accumulate in the TME, this characteristic enables them to be used as targeted carriers for tumor treatment, which can deliver therapeutic agents directly to tumors, enhancing efficacy and reducing systemic side effects [247]. As a tumor-targeting system, non-pathogenic *E. coli* strain MG1655 can deliver TNF-α specifically in tumor sites, thereby localizing its tumor-killing effect and achieving precise control over the targeted therapy [248].

Compared to non-engineered bacteria, engineered bacteria offer greater clinical potential by delivering anti-tumor agents, expressing tumor-specific antigens, or activating immune responses, thus improving the precision and effectiveness of tumor therapies. Firstly, engineered bacteria can modulate immunity by expressing tumor antigens or inducing the release of immune regulatory factors. For instance, both *Listeria monocytogenes* and *Salmonella enterica* can deliver inherently expressed proteins as tumor antigens to antigen-presenting cells, thereby activating anti-tumor immunity [249]. Additionally, incorporating an immunoregulatory factor, α-galactosylceramide, into engineered *Listeria monocytogenes* can significantly activate NKT cells, reduce metastatic lesions, and concurrently control cytotoxicity [250]. Moreover, some engineered microbiomes can directly perform anti-tumor effects once equipped with therapeutic agents. An attenuated live *Listeria monocytogenes*, once coupled with the radioisotope rhenium, could be endowed with radioactive characteristics and accurately eliminate tumor tissue without damaging normal tissues in metastatic pancreatic cancer [251]. Moreover, another study proposed a new strategy to enhance bacterial metabolic activity through nano photocatalysts. By combining carbon–nitrogen compounds with *E. coli*, they can promote bacterial production of nitric oxide with anti-tumor effects under light irradiation, significantly improving treatment efficiency [252]. However, a stable bacterial carrier is required to periodically release agents to achieve clinical application. Din et al. developed a type of engineered bacterial community that will synchronously lyse and release drug payloads encoded by genes when the population density reaches a threshold [253]. A small number of surviving bacteria will reproduce again, forming pulsed drug delivery, this engineered bacterium can reduce tumor activity and significantly improve the survival rate of mice when used in combination with chemotherapy [253]. Engineered *Salmonella* can also serve as an anti-tumor treatment vector, gradually releasing therapeutic Cp53 peptides in response to tetracycline, thereby inducing host cell death [254].

**Fig. 7:**
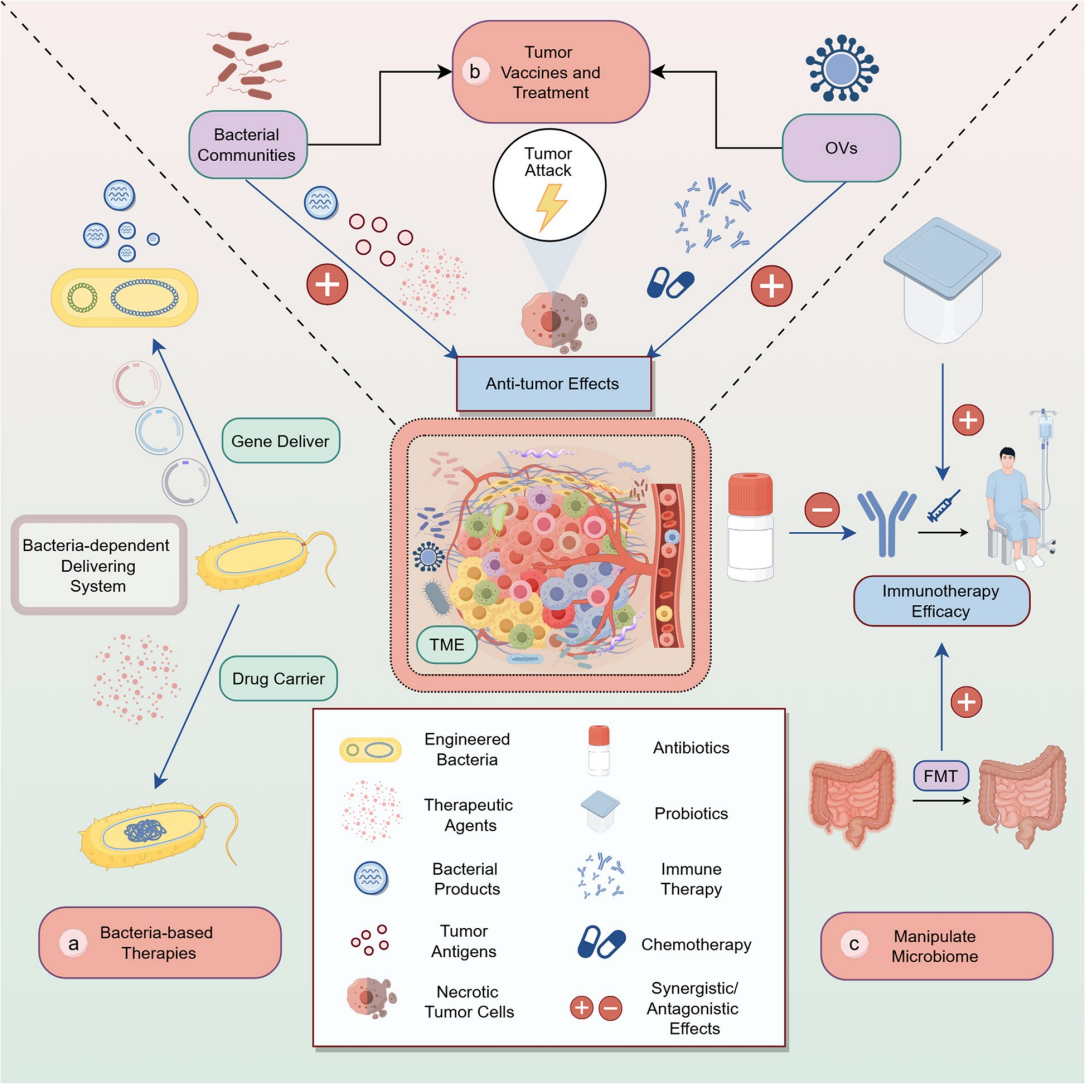
Microbial-based interventions as strategies for enhancing anti-tumor effects and improving immunotherapy efficacy. **a** Bacteria-based therapies, bacteria-dependent delivering system involves gene deliver and drug carrier, bacterial products derived from gene-modified bacteria and therapeutic agents secreted by bacterial carriers can exhibit anti-neoplastic effects; **b** Tumor Vaccines and Treatments, both bacteria and OVs play important roles in activating anti-tumor effects through expressing tumor antigens, lysing tumor cells, and enhancing tumor modalities; **c** Manipulating Microbiome, FMT and probiotics have the potential to enhance immunotherapy efficacy, whereas antibiotics may impair immune therapy responsiveness. (Generated using Figdraw)

In addition to delivering therapeutic agents to tumor sites, some of bacteria could also carry specific genes after being engineered, that is, transferring plasmid DNA into mammalian cells through bacteria for the expression of exogenous proteins [255]. Specific genes fragments were integrated into tumor tissues, would consequently play corresponding anti-tumor effects, depending on specific circumstances. Notably, other potential methods could involve leveraging the immune system. In concordance with previous findings, CD47-induced tumor immune evasion can be targeted by engineering non-pathogenic *E. coli* to specifically lyse and release anti-CD47 nanobodies in the TME, enhancing the activity of tumor-infiltrating T cells, promoting tumor regression, and preventing metastasis [139]. In addition, local injection of this engineered bacterium can trigger a systemic immune response, reduce the growth of untreated tumors, and demonstrate an ‘abscopal effect’ [139]. General mechanisms on microbial-based interventions are illustrated in Fig. 7.

### OVs

OVs, classified into single-stranded RNA, double-stranded DNA, and other types, have received widespread attention in the twenty-first century due to their ability to directly lyse tumors and activate immune responses [256]. Likewise, the in situ vaccination effects of OVs can bolster anti-tumor defenses. Concurrently, OVs can modulate the TME, inhibit tumor angiogenesis and metabolism, and have the potential to further enhance anti-tumor effects when combined with chemotherapy and immunotherapy [256].

Specifically, certain viruses have also been utilized as the agent of boosting the host immunity for the purpose of tumor treatment, such as herpes simplex virus, adenovirus, vaccinia virus, and reovirus [256]. Talimogene laherparepvec (T-VEC), a genetically modified type I herpes simplex virus that specifically replicates in tumor cells, is the only OVs currently approved by food and drug administration (FDA) for treating advanced melanoma [257]. T-VEC showed a significant therapeutic effect in phase III clinical trials for unresectable stage IIIB-IV melanoma, demonstrating higher persistent response rates and overall survival (OS) rates than human granulocyte–macrophage colony-stimulating factor (GM-CSF), particularly in early and untreated patients, with tolerable side effects [257]. It triggers local and systemic immune responses through intralesional delivery, leading to tumor cell lysis, release of tumor antigens, and activation of specific effector T cells [258]. Besides, GM-CSF gene is inserted into T-VEC to promote local GM-CSF production, enhance the recruitment and activation of DCs, and thus initiate systemic anti-tumor responses [259].

OVs also offer flexibility by introducing key immune factors into TME, making them an important tool in combination therapy. Research has shown that combining OVs with ICIs, adoptive cell therapy (ACT), chemotherapy, or targeted drugs yields promising results. This combinational therapy boosts efficacy in patients with immunosuppressive status. For instance, CF33 is an effective recombinant orthopoxvirus backbone targeting CRC, it was detected that CF33 infection upregulated PD-L1 expression in tumor cells and increased lymphocytes and macrophage infiltration in the TME [260]. Additionally, the combination of ACT and OVs has gained attention for its synergistic anti-tumor effects. ACT is an effective method of genetically modifying T cells or NK cells of patients in vitro before reintroducing them back into the human body. The main types include tumor infiltrating lymphocytes (TILs), chimeric antigen receptor T-cell therapy (CAR-T), T-cell receptor engineered T-cell therapy (TCR-T), and NK cell therapy [261]. For instance, studies show that combining NKT cell immunotherapy with the recombinant oncolytic virus VSV-IL-15 and PD-1 blockade can enhance tumor control in pancreatic cancer and prolong survival, demonstrating a promising therapeutic strategy [262]. In terms of CAR-T therapy, by constructing recombinant tumor targeted adenovirus expressing CD19 tags, tumors with different antigens can be labeled as a single anti-CD19 CAR-T recognition target, which can become a powerful supplement to anti antigen mismatched solid tumor immunotherapy [263]. Also, tumor cells will transform into artificial antigen-presenting cells when infected by herpes simplex virus type 1 (HSV-1) based OVs encoding OX40 ligand and IL-12, then the effectiveness of TIL therapy will be reinforced under the combination of OV-OX40L/IL12 and TILs therapy, achieving complete tumor regression, triggering antitumor immune memory, and reprogramming immune cells in TME [264]. Besides, OVs therapy will also exhibit augmented efficacy when combined with chemotherapeutics. May V et al. explored the effect of measles vaccine virus therapy combined with gemcitabine in the treatment of advanced pancreatic cancer, reducing tumor mass by more than 50% [265]. Additionally, TG4010, a modified ankara vaccinia virus expressing Mucin 1 and IL-2, significantly extended progression-free survival when combined with chemotherapy compared to placebo plus chemotherapy [266]. Clinical trials concerning oncoviruses and their combination with other tumor therapies are listed in Table 3. The above effects demonstrated the prospect combining OVs with other promising tumor therapies, illuminating the fact that numerous ongoing clinical trials are still needed to further explore and optimize these modalities.

**Table 3:** Recent clinical trials on ovs therapy and its combinations with other tumor treatment modalities

OVs Type	Treatment Modality (Experimental Group)	Malignancy Type	Clinical Outcome	NCT Number	Ref
Adenovirus	NG-350A	Advanced/Metastatic Cancer	Demonstrate tumor delivery, viral replication, and transgene expression via intravenous and intratumoral administration without transgene-related or off-target toxicity	NCT05165433, NCT06459869	[267]
Adenovirus	YSCH-01	Advanced Solid Tumors	Demonstrate that YSCH-01 (recombinant L-IFN adenovirus) is a safe, well-tolerated treatment for advanced solid tumors, with promising preliminary efficacy that warrants further investigation to confirm its effectiveness and safety	NCT05180851	[268]
Adenovirus	LOAd703 with nab-paclitaxel plus gemcitabine	Advanced PDAC	Combined modality is feasible and safe in cancer treatment	NCT02705196	[269]
Adenovirus	TILT-123	Advanced Solid Cancers	Generate antitumor effects in both local and distant lesions with good tolerability and safety, making it suitable for combination therapy	NCT04217473, NCT05271318, NCT05222932, NCT06125197	[270]
Adenovirus	Ad5-IL-12 Adenovirus	Prostate Cancer	Possess remarkable tolerance for local delivery and can activate immune responses	NCT02555397	[271]
Adenovirus	AdAPT-001	Advanced Solid Tumors	Show a good tolerability and anti-tumor effects in a suitable dose administered every two weeks	NCT04673942	[272]
Adenovirus	OBP-301	Advanced Hepatocellular Carcinoma	Show an increased CD8 + T-cell infiltration and localized necrosis at injection sites, with well tolerability and potential for improved efficacy in combination with other immunotherapies	NCT02293850	[273]
Adenovirus	ONCOS-102	Malignant Solid Tumour	Demonstrate safety, tolerability, and evidence of antitumor immunity at all tested doses, with potential as an immunosensitizing agent for combination therapies with immunotherapy	NCT01598129	[274]
Adenovirus	ONCOS-102 plus pembrolizumab	Advanced Melanoma	The combination therapy of ONCOS-102 and pembrolizumab can significantly inhibit melanoma growth	NCT03003676	[275]
Adenovirus	Cretostimogene Grenadenorepvec plus pembrolizumab	NMIBC	Show a durable efficacy and a favorable safety profile in patients with BCG-unresponsive NMIBC	NCT04387461	[276]
Adenovirus	VB-111 plus nivolumab	Metastatic CRC (Microsatellite stable)	Exhibit minimal toxicities without exhibiting the overall response rates or survival	NCT04166383	[277]
Adenovirus	DNX-2401 plus pembrolizumab	Glioblastoma	Provide survival benefits in some patients with a good tolerability, despite the objective response rate not meeting expectations	NCT02798406	[278]
Adenovirus	Enadenotucirev plus nivolumab	Advanced Epithelial Cancer	Show a manageable tolerability, promising OS, with inducing immune cell infiltration	NCT02636036	[279]
Herpes Simplex Virus Type 1	T-VEC plus neoadjuvant chemotherapy	TNBC	Show a promising results, with a 45.9% RCB0 rate and 65% RCB0-1 rate, and this modality is well tolerated	NCT02779855	[280]
Herpes Simplex Virus Type 1	T-VEC plus ipilimumab	Advanced Melanoma	The combined modality significantly enhance durable response rates over ipilimumab alone, with no additional toxicity after the 5-year follow-up	NCT01740297	[281]
Herpes Simplex Virus Type 1	T-VEC plus atezolizumab	TNBC and Metastatic CRC	Exhibit a manageable safety profile, with no unexpected risks, but showed limited antitumor activity	NCT03256344	[282]
Herpes Simplex Virus Type 1	G207	Recurrent or Progressive Glioma	Demonstrate an acceptable adverse event profile and show responses both alone and combined with radiation	NCT02457845	[283]
Herpes Simplex Virus Type 1	T-VEC plus pembrolizumab	Advanced Melanoma	T-VEC combined with pembrolizumab demonstrate an antitumor activity and tolerability in PD-1-refractory melanoma, particularly in patients with disease recurrence during or after adjuvant anti-PD-1 therapy	NCT04068181	[284]
Herpes Simplex Virus Type 2	oHSV2	Melanoma	Ultraviolet-oHSV2 has anti-tumor effects by activating NK cells through TLR2/NF-κB, with the potential for enhancing therapeutic efficacy by combining anti-NKG2A and anti-HLA-E antibodies	NCT03866525	[285]
Vaccinia Virus	Olvi-vec	Malignant pleural mesothelioma or Metastatic NSCLC or breast cancer	Demonstrate the safety and feasibility while enhancing local immune responses and increasing immune cell and protein expression in patients	NCT01766739	[286]
Vaccinia virus	Pexa-Vec plus durvalumab and tremelimumab	Metastatic CRC	Show a safe and tolerable trait and potential clinical activity in pMMR CRC patients, with no unexpected toxicities	NCT03206073	[287]
Coxsackievirus A21	V937 plus pembrolizumab	Advanced Solid Tumors	Show manageable safety but lack superior efficacy over immunotherapies in NSCLC and urothelial cancer	NCT02043665	[288]
Recombinant Polio-Rhinovirus	PVSRIPO	Recurrent Glioma	Higher survival rates at 24 and 36 months	NCT01491893	[289]
Reovirus	Pelareorep combined with carboplatin and paclitaxel	Metastatic PDAC	Combination therapy doesn’t improve progression-free survival, targeting immunosuppressive factors may enhance efficacy based on immunophenotypes	NCT01280058	[290]
Measles virus	MV-CEA	Recurrent Glioblastoma	Exhibit a high potential in the treatment with a reliable safety	NCT00390299	[291]

*Abbreviations*: *RCB0* no residual cancer after treatment, representing a complete response, *RCB1* minimal residual cancer, reflecting a very favorable response to therapy, *RCB 0–1* RCB0 or RCB1, *NKG2A* natural killer group 2 member A, *HLA-E* human leukocyte antigen E, *pMMR* standard chemotherapy refractory mismatch repair proficient

### Manipulate tumor microbiome to enhance antitumor efficacy

Studies have demonstrated that microbiota can influence the effectiveness of various tumor therapies, including chemotherapy, immunotherapy, and targeted therapies. Therefore, targeting specific microbial communities may enhance tumor treatment efficacy. Microbiota-based interventions, due to their role in immune regulation, have shown great potential to influence patients' prognosis through immunomodulatory effects in clinical trials, including antibiotics, probiotics, and fecal microbiota transplantation (FMT).

Anti-tumor antibiotics are important treatments that exhibit specific inhibitory effects on the proliferation and metastasis of tumor cells. They primarily inhibit tumor development by suppressing tumor proliferation, inducing apoptosis, and repressing EMT [292]. Specifically, itraconazole has anti-tumor activity in breast cancer cells. It induces apoptosis by altering mitochondrial membrane potential, reducing B-cell lymphoma-2 (BCL-2) expression, and increasing caspase-3 activity, indicating significant anti-tumor effects [293]. Another study indicates that itraconazole also has anti-tumor effects on oral squamous cell carcinoma by inhibiting the Hedgehog signaling pathway [294]. However, antibiotic administration can also disrupt gut microbiota, impairing patients' responsiveness to immunotherapy. For instance, a clinical trial evaluated the impact of antibiotic exposure on the clinical outcomes of non-small cell lung cancer (NSCLC) patients receiving anti-PD(L)−1 inhibitors combined with platinum. Patients who received antibiotics within 60 days prior to treatment had poorer outcomes, including lower objective response rates (ORR), shorter progression-free survival (PFS), and reduced OS [295]. Similarly, an observational study also suggested that tumor patients who receive antibiotic therapy before ICI treatment have shorter OS and poorer response to ICIs, while simultaneous antibiotic therapy has no significant effect on efficacy [296]. However, in pancreatic cancer, a study shows that eliminating microorganisms can inhibit tumor development, reverse immune tolerance, and enhance the effectiveness of immune checkpoint therapy [19]. Moreover, Pushalkar S et al. proposed the upregulation of PD-1 expression levels after removing bacterial communities, which enhances the efficacy of ICIs. Mechanistically, the microbiome of pancreatic cancer produces a tolerant immune program by activating specific TLRs, leading to T cell dysfunction [19]. Therefore, to understand the complexity of the TME and antibiotic therapy, further research is needed to effectively improve treatment efficacy.

Currently, probiotic supplementation is also considered an effective strategy to improve anti-tumor efficacy. The most widely acknowledged bacterial populations are *Lactobacillus* and *Bifidobacterium.* Probiotic-mediated immunomodulation is based on their propensity to inhibit the production of bone marrow-derived neutrophils, which are responsible for enhancing metastasis in response to tumor-derived stimulation [297]. Moreover, at the cellular level, the probiotics *Lactobacillus casei* and *Lactobacillus rhamnosus* have been shown to inhibit colon cancer metastasis by regulating MMP-9 expression. Given the role of MMPs in promoting cancer metastasis through ECM dissolution, it is reasonable to conclude that reducing MMP expression would counteract cancer cell invasion [298]. From the perspective of cellular adhesion, live *Lactobacilli* inhibit cervical cancer cells by upregulating E-cadherin [299]. Similar effects of E-cadherin upregulation were observed, significantly reducing metastasized lung lesions [300]. *Bifidobacterium*, another crucial probiotic, can also enhance immunotherapy by infiltrating the TME in a process dependent on STING [138]. Remarkably, this process can enhance antigen presentation by DCs and strengthen the tumor-eliminating effects of CD8 + T cells [138]. In conjunction with probiotics, dietary interventions also play a significant role in immune function. Dietary modification is widely recognized as an adjunct to antineoplastic therapy with high patient compliance and acceptance [301]. Sundaram S et al. demonstrated the effects of dietary modification on breast cancer prognosis. By adding selenium in the form of methylseleninic acid to the daily consumption, the decreased tumor load and lung metastatic foli were observed in MMTV-PyMT mice [302]. The Mediterranean diet, rich in nutrients with antioxidant and anti-inflammatory properties, has been utilized as an effective adjuvant, showing inhibitory effects on metastasis and proliferation in breast cancer when administered routinely [303]. Considering its accessibility, it is crucial to apply this supplemental diet as an effective method of both resisting tumor recurrence and improving patients prognosis [304]. In alignment with other prevalent therapies, vitamin D supplementation also plays a pivotal role in reducing the incidence of pancreatic cancer metastasis by inactivating tumor-associated fibroblasts [305]. Dietary habit modifications are increasingly recognized as an indispensable adjuvant therapy to suppress metastasis, improve prognosis, and enhance quality of life.

FMT is ubiquitously used in treating refractory colitis, including C*lostridium difficile*-associated colitis and immune-related colitis [306]. In recent years, FMT has also been increasingly recognized for its immunomodulatory effects and has been used as a potential treatment in multiple metastatic malignancies, especially melanoma [307–309]. Specifically, Baruch et al. and Davar et al. were the first to confirm that FMT may improve the response of metastatic melanoma patients to PD-1 immunotherapy. They found that with increased abundance of specific microbiota, CD8 + T cell activation was enhanced, and immune-suppressive myeloid cells decreased [10]. Moreover, Gopalakrishnan et al. revealed that FMT from ICI-responsive melanoma patients reduced tumor growth in mice in an immune cell-dependent manner. In contrast, FMT from patients who did not respond to ICIs did not demonstrate this effect. Tumor microbes have unique immunomodulatory capabilities and offer wide-ranging potential applications in tumor therapy through their interactions with the host immune system.

Due to their unique immunomodulatory capabilities, the composition and dynamic characteristics of microbiota are increasingly recognized as potential biomarkers to predict patients' response to immunotherapy and tumor progression, providing a basis for personalized treatment. Therefore, using microbiota as diagnostic and therapeutic biomarkers in tumor treatment is expected to further optimize treatment regimens and improve patient survival. To better highlight the current clinical landscape, recent registered clinical trials focused on microbiome manipulation involving probiotics, antibiotics, and FMT are listed in Table 4.

**Table 4:** Recent clinical trials on the impact of microbiome-based modulation of immunotherapy efficacy

Microbial Modulation	Treatment Modality (Experimental Group)	Malignancy Type	Clinical Outcome	NCT Number	Ref
Antibiotics, Probiotics (SER-401)	Vancomycin pretreatment plus SER-401 and nivolumab	Metastatic Melanoma	Vancomycin preconditioning with SER-401 and anti-PD-1 is safe in metastatic melanoma, though antibiotics may impair immune response	NCT03817125	[310]
Antibiotics	Antibiotic therapy plus atezolizumab	Urothelial Carcinoma	Contribute to worse survival outcomes	NCT02108652, NCT02302807	[311]
Antibiotics	Antibiotic therapy atezolizumab	NSCLC	Exhibit short OS significantly	NCT01903993, NCT02008227	[312]
Antibiotics	Antibiotic therapy plus pembrolizumab	Muscle-invasive Bladder Cancer	Exhibit lower rates of complete response and of recurrence-free survival	NCT02736266	[313]
Probiotics	SYNB1891 (Engineered *E. coli Nissle* 1917) plus atezolizumab	Refractory Advanced Cancers	Show strong safety, tolerability, and STING pathway activation, achieving disease stability in some PD-1/L1-resistant patients	NCT04167137	[314]
Probiotics	CBM588 (*Bifidogenic* live bacterial product) plus nivolumab and ipilimumab	Metastatic Renal Cell Carcinoma	Significantly prolong PFS of patients	NCT03829111	[315]
FMT	FMT plus anti-PD-1	Advanced Melanoma	Effective in some PD-1-resistant melanoma patients by altering gut microbiota, boosting immune response, and overcoming anti-PD-1 resistance	NCT03341143	[9]
FMT	FMT plus PD-1 inhibitors	Advanced Melanoma	Investigate converting potential of FMT in immunotherapy responses	NCT05251389	[316]

## Conclusive remarks

Overall, the discovery of tumor microbiome over the past few decades has introduced new perspectives and potential therapeutic targets for tumor treatment. Growing research demonstrates that these microbiota not only reflect the tumor's pathological type, drug response, and prognosis, but also play biological roles at various stages of tumor initiation, development, and metastasis. Specifically, the microbiota can contribute to carcinogenesis through activating specific pathways, inducing DNA damage, modifying epigenetic profiles, and promoting chronic inflammation. Additionally, the tumor microbiome can influence angiogenesis and immune responses, facilitating tumor progression through intrinsic and extrinsic modifications that promote metastasis. In the clinical landscape, microbiome-based therapies can be utilized to enhance the efficacy of chemotherapy, immunotherapy, and even radiotherapy. Cutting-edge therapeutic options employ oncolytic viruses and engineered bacteria to enable targeted drug delivery, the release of cytotoxic agents, and enhancement of anti-tumor immunity.

However, despite the promising future of the tumor microbiota, the comprehensive understanding of its role in tumor progression still remains limited. This primarily arises from insufficient experimental tools for tracking the spatial dynamics and activities of these microbes, accurately interfering with their functions, and effectively manipulating the microbial genome for mechanistic studies. Additionally, intratumor microbiota has not been clearly defined in some studies and is often confounded by other microbial sources, making it difficult to distinguish between intratumoral and tumor microorganisms. Therefore, future studies should focus on the origin of microorganisms, their interactions with tumor cells, and their functional and physiological significance in the TME to drive technological advances and improve experimental approaches.

In conclusion, the tumor microbiota deserves in-depth study as a key determinant of tumor pathogenesis and treatment response. Although multiple challenges remain, through interdisciplinary collaboration, technological innovation, and strict ethical standards, the study of tumor microbiota is expected to drive breakthroughs in tumor therapy, leading to better prognoses and therapeutic outcomes for patients.

## Data Availability

Not applicable.
